# *Porphyromonas gingivalis* potentiates stem-like properties of oral squamous cell carcinoma by modulating SCD1-dependent lipid synthesis via NOD1/KLF5 axis

**DOI:** 10.1038/s41368-024-00342-8

**Published:** 2025-02-28

**Authors:** Wenli Zang, Fengxue Geng, Junchao Liu, Zengxu Wang, Shuwei Zhang, Yuchao Li, Ze Lu, Yaping Pan

**Affiliations:** 1 Department of Periodontics, School and Hospital of Stomatology, China Medical University, Liaoning Provincial Key Laboratory of Oral Disease, Shenyang, China; 2 Department of Oral Maxillofacial-Head and Neck Surgery, School and Hospital of Stomatology, China Medical University, Liaoning Provincial Key Laboratory of Oral Disease, Shenyang, China; 3 Department of Periodontics and Oral Biology, School and Hospital of Stomatology, China Medical University, Liaoning Provincial Key Laboratory of Oral Disease, Shenyang, China

**Keywords:** Microbiology, Oncology

## Abstract

Cancer stem cells (CSCs) are widely acknowledged as primary mediators to the initiation and progression of tumors. The association between microbial infection and cancer stemness has garnered considerable scholarly interest in recent years. *Porphyromonas gingivalis* (*P. gingivalis*) is increasingly considered to be closely related to the development of oral squamous cell carcinoma (OSCC). Nevertheless, the role of *P. gingivalis* in the stemness of OSCC cells remains uncertain. Herein, we showed that *P. gingivalis* was positively correlated with CSC markers expression in human OSCC specimens, promoted the stemness and tumorigenicity of OSCC cells, and enhanced tumor formation in nude mice. Mechanistically, *P. gingivalis* increased lipid synthesis in OSCC cells by upregulating the expression of stearoyl-CoA desaturase 1 (SCD1) expression, a key enzyme involved in lipid metabolism, which ultimately resulted in enhanced acquisition of stemness. Moreover, SCD1 suppression attenuated *P. gingivalis*-induced stemness of OSCC cells, including CSCs markers expression, sphere formation ability, chemoresistance, and tumor growth, in OSCC cells both in vitro and in vivo. Additionally, upregulation of SCD1 in *P. gingivalis*-infected OSCC cells was associated with the expression of KLF5, and that was modulated by *P. gingivalis-*activated NOD1 signaling. Taken together, these findings highlight the importance of SCD1-dependent lipid synthesis in *P. gingivalis-*induced stemness acquisition in OSCC cells, suggest that the NOD1/KLF5 axis may play a key role in regulating SCD1 expression and provide a molecular basis for targeting SCD1 as a new option for attenuating OSCC cells stemness.

## Introduction

Epidemiological studies highlight that microorganisms are widely present in cancer tissues and closely related to the occurrence, development and prognosis of cancers.^1^ Accumulating evidence suggests that oral bacteria is a potential risk factor for the occurrence and development of Oral squamous cell carcinomas (OSCC).^2,3^ A meta-analysis indicated that periodontal pathogens increased the incidence of cancers including OSCC, and was related to poor overall survival, disease-free survival, and cancer-specific survival.^4^ *Porphyromonas gingivalis* (*P. gingivalis*), one of the most well-studied periodontal pathogens, is strongly associated with the tumorigenesis and progression of OSCC.^5–8^ Our and other previous studies have reported that the relative abundance of *P. gingivalis* was elevated in OSCC tissues and was associated with shorter overall survival.^9,10^ *P. gingivalis* can manipulate the host immune system,^9^ induce chronic inflammation,^7^ and accelerate the proliferation and metastatic dissemination of OSCC cells.^11^

These years, studies reveal that the poor prognosis of OSCC patients is associated with a cell subpopulation called cancer stem cells (CSCs).^12^ CSCs constitute a rare but important subpopulation in a variety of tumors with self-renewal, multilineage differentiation, and limitless proliferation abilities. Growing evidence has shown that CSCs are considered to be responsible for tumor initiation, progression, and chemoresistance.^13^ Recently, studies have indicated that non-CSCs can acquire CSC features such as expressing CSC markers, efficiently forming tumorspheres in vitro, and forming tumors in vivo by interacting with the surrounding tumor microenvironment.^14^ Notably, several kinds of gram-negative bacteria, such as *Fusobacterium nucleatum* and *Helicobacter pylori*, are reported to drive cancer cell to acquire stem-like properties.^15,16^ Previous studies have confirmed that *P. gingivalis* could promote epithelial-mesenchymal-transition phenotype of OSCC cells, which is an important process to induce cancer stemness.^8,17^ Na Hee Ha et al. preliminarily explored that *P. gingivalis* could induce OSCC cells to highly express two common CSC markers, CD44 and CD133.^18^ However, the evidence that *P. gingivalis* can affect the stemness of OSCC cells has yet to be determined.

Metabolic reprogramming is considered a hallmark by which cancer cells to sustain high proliferation rates and satisfy energy requirements. In addition to high levels of glycolysis (Warburg effect) in cancer cells, alterations in lipid metabolism play crucial roles in cancer development and progression.^19^ Recently, studies have shown that lipid metabolism alterations modulate the generation and maintenance of CSCs properties by balancing lipid biosynthesis and catabolism.^20^ Specifically, excess de novo lipids are stored in the form of lipid droplets (LDs), which are targetable features of CSCs.^21^ LDs are intracellular organelles that accumulate highly in CSCs in numerous types of cancers, such as colorectal cancer, pancreatic cancer, and breast cancer.^22,23^ Importantly, Tirinato et al. demonstrated that sorted colorectal CSCs with high LDs levels form tumors faster than CSCs with a low amount of LDs do, supporting a direct correlation between increased LD levels and increased tumorigenic potential.^24^ Moreover, LDs are proposed as functional markers of CSCs; they not only sustain lipid and energy homeostasis but also activate important stemness signaling pathways.^16,24^ Stearoyl-CoA desaturase 1 (SCD1) is a key regulator of lipid metabolism located in the endoplasmic reticulum (ER) and functions as a desaturase to convert saturated fatty acids to monounsaturated fatty acids, which are the preferred substrates for lipid synthesis.^25^ Studies have shown that SCD1 is upregulated in multiple types of cancers and is important for cancer progression and stemness properties.^26^ Thus, there is an urgent need to explore whether lipid metabolism disorders are involved in stemness acquisition in OSCC cells.

Krüppel-like factor 5 (KLF5) is a member of the KLF family of zinc finger transcription factors. KLF5 is normally considered protumorigenic, and previous studies have shown that KLF5 is highly expressed in various cancer types and contributes to cancer metastasis and development.^27^ Moreover, KLF5 can regulate multiple cellular functions, including proliferation, differentiation, migration, stemness, and tumorigenesis, by regulating downstream target genes.^28^ Recently, KLF5 was reported to be strongly correlated with energy metabolism in several tissues.^29^ For example, KLF5 can bind to sterol-regulatory-element-binding protein-1 (SREBP-1) to activate fatty acid synthase (FASN) transcription, thereby increasing the proliferation of prostate cancer cells.^30^ KLF5 interacts with SREBP-1 and TP63 to regulate lipid metabolism in SCC cells.^31^ Additionally, KLF5 coregulates peroxisome proliferator-activated receptor-delta (PPAR-δ) and acts as a molecular switch in lipid metabolism in skeletal muscle.^32^ Although several mechanisms by which KLF5 is involved in lipid metabolism have been reported, whether KLF5 is involved in the acquisition of stem cell-like features via lipid metabolism remains unclear.

In this study, we explored the potential association between *P. gingivalis* and the stemness properties of OSCC cells via clinical tissue samples, a cellular model of persistent infection with *P. gingivalis*, and an in vivo xenotransplantation model. Through high-throughput sequencing, bioinformatics analysis, and related mechanism studies, SCD1-dependent lipid synthesis via the NOD1/KLF5 axis was identified as a key pathway involved in regulating OSCC cell stemness during *P. gingivalis* infection. These findings reveal a promising target for improving OSCC treatment outcomes.

## Results

### *P. gingivalis* positively correlated with the expression of CSCs markers in OSCC samples

**Fig. 1:**
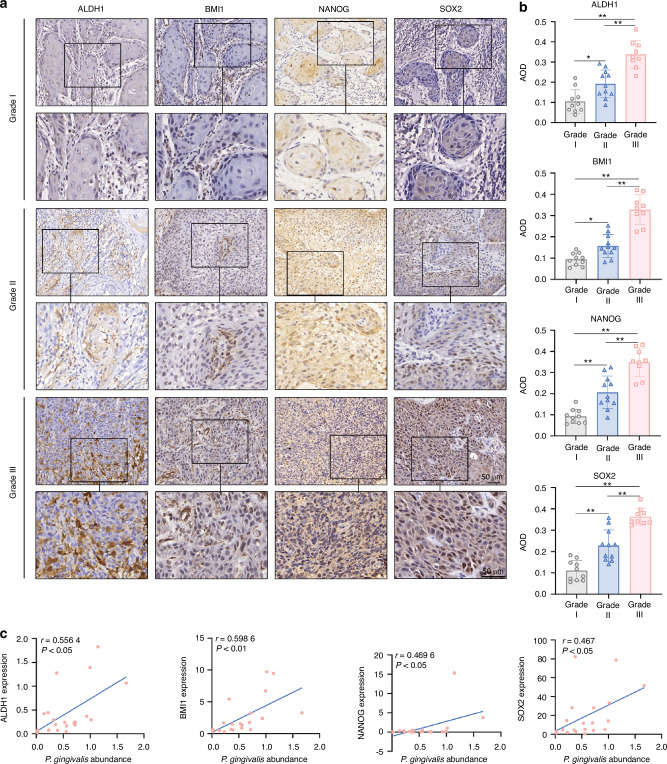
The correlation of *P. gingivalis* abundance and cancer stem cell markers expression in OSCC tissues. **a** Representative IHC images of ALDH1, BMI1, NANOG, and SOX2 in OSCC samples. Scale bar: 50 μm. **b** Quantitative analysis of ALDH1, BMI1, NANOG, and SOX2 in OSCC samples. *n* = 30. Data are presented as mean ± SD. *P < 0.05, **P < 0.01. **c** The correlations between *P. gingivalis* and the expression of ALDH1, BMI1, NANOG, and SOX2 were analyzed with Pearson correlation analysis. *n* = 20

To determine key CSC markers involved in OSCC progression, we carried immunohistochemical staining of human OSCC samples. We showed the expression levels of NANOG, BMI1, and SOX2, which are the pluripotency transcription factors involved in the regulation of CSCs, strongly grew along with advanced pathological tumor grade, whereas OCT4 was unchanged (Fig. 1a, b and Supplementary Fig. 1a, b). Similarly, the protein level of ALDH1, a functional marker of CSC, were also correlated with OSCC progression (Fig. 1a, b). Our previous study has indicated that *P. gingivalis* is enriched in OSCC tissues and positively associated with late clinical staging, low differentiation, and lymph node metastasis in OSCC patients.^10^ Hence, we further investigated the relative amount of *P. gingivalis* and the expression of these CSC markers in twenty OSCC cancerous tissues by qRT-PCR assay to explore whether there was an association between *P. gingivalis* and CSC markers. Pearson correlation analysis results showed that the amount of *P. gingivalis* positively correlated with the expression levels of ALDH1, SOX2, NANOG, and BMI1 (*P* < 0.05) (Fig. 1c and Supplementary Fig. 1c). In order to further explore whether the effect of *P. gingivalis* on the stemness of OSCC was specific, we further examined the correlation between the *F. nucleatum* and stemness markers in OSCC cancerous tissues. The results showed there was no correlation between the abundance of *F. nucleatum* and the gene expression levels of CSC markers (*P* > 0.05) (Supplementary Fig. 1d). Consequently, our data strongly suggested that *P. gingivalis* played an essential role in the stemness maintenance of OSCC through specifically expressing ALDH1, SOX2, NANOG, and BMI1.

### Persistent exposure to *P. gingivalis* induced the stemness of OSCC cells

**Fig. 2:**
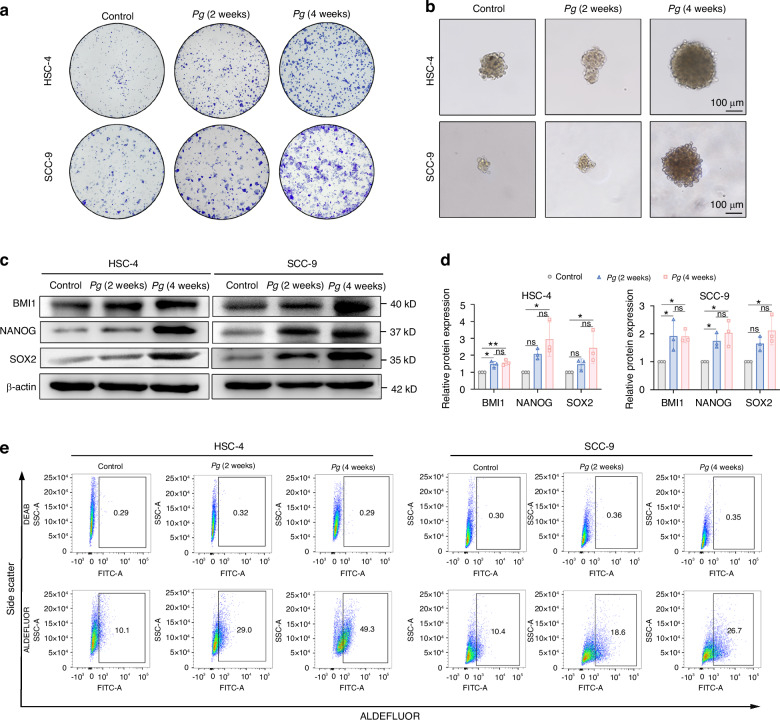
Persistent exposure to *P. gingivalis* promoted OSCC cells to acquire stem-like features. **a** Colony formation showed *P. gingivalis* increased the number of cell clones. **b** Sphere formation showed *P. gingivalis* increased the diameter of tumorspheres. Scale bar: 100 μm. **c**, **d** Western blot and quantification showed the upregulation of stemness signature proteins BMI1, NANOG, and SOX2 compared to the control group. β-actin was used as a housekeeping gene. **e** Flow cytometry showed *P. gingivalis* increased the proportion of ALDH1^+^ subpopulation of HSC-4 and SCC-9 cells. *n* = 3. Data are presented as mean ± SD. ns: *P* > 0.05, *P < 0.05, ** *P* < 0.01

To explore whether *P. gingivalis* infection can induce the stemness of OSCC cells, we established a persistent *P. gingivalis* infection model of HSC-4 and SCC-9 cells and the colonies obtained indicated that *P. gingivalis* could remain viable during long-term infection (Supplementary Fig. 2). Meanwhile, the results showed that the viability of *P. gingivalis*-infected OSCC cells was significantly increased with a MOI of 10 in a time-dependent manner (Supplementary Fig. 3a–c). Similarly, co-culture with *P. gingivalis* promoted cell migration, invasion, and colony formation abilities compared with the control cells (Fig. 2a and Supplementary Fig. 3d–h). Moreover, *P. gingivalis* significantly enhanced spheroid formation ability reflected by the number and diameter of tumorspheres in both HSC-4 and SCC-9 cells (Fig. 2b and Supplementary Fig. 3i). We next further evaluated the impact of *P. gingivalis* on the expression of stemness signature proteins SOX2, NANOG, and BMI1. As expected with the phenotypes, the expression of these markers were notably increased after persistent exposure to *P. gingivalis* (Fig. 2c, d). Consistent with the above results, flow cytometry also showed that the higher ratios of the ALDH1^+^ subpopulation in *P. gingivalis*-treated OSCC cells than the respective control (Fig. 2e and Supplementary Fig. 3j). Taken together, these results revealed that *P. gingivalis* promoted CSCs properties of OSCC cells in vitro. Additionally, in order to further explore the effects of *P. gingivalis* on stemness dependent on which bacterial components, we added heat-inactivated *P. gingivalis*, gingipains, and lipopolysaccharide (LPS) groups. Interestingly, heat-inactivated *P. gingivalis*, gingipains, and LPS could not cause significant changes in the expression of stemness markers (Supplementary Fig. 4a–d), suggesting that *P. gingivalis* induced stemness of OSCC cells may depend on live whole condition.

### *P. gingivalis* enhanced lipid synthesis to promote the stemness of OSCC cells based on bioinformatical analysis

**Fig. 3:**
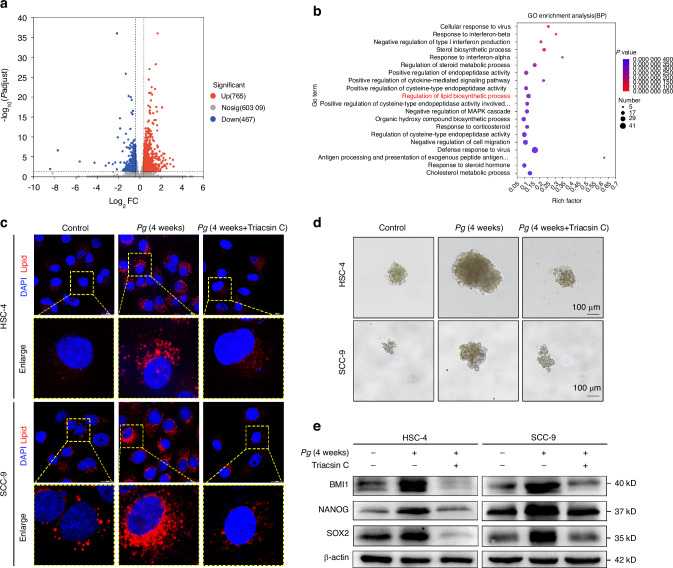
*P. gingivalis* induced OSCC cells to acquire stem-like features by regulating lipid synthesis. **a** Volcano plot of differentially expressed genes (DEGs) between HSC-4 cells treated with and without *P. gingivalis*. **b** Top twenty most enriched GO (BP) pathways enrichment analysis for differential genes were visible. **c** Nile red staining showed Triacsin C abolished *P. gingivalis*-induced lipid droplets accumulation. Scale bar: 20 μm. **d** Sphere formation showed Triacsin C decreased *P. gingivalis*-induced the diameter of tumorspheres. Scale bar: 100 μm. **e** Western blot showed Triacsin C significantly suppressed *P. gingivalis*-induced upregulation of BMI1, NANOG, and SOX2. β-actin was used as a housekeeping gene. *n* = 3

We performed high-throughput sequencing analysis to determine differentially expressed transcriptomic genes in HSC-4 cells co-cultured with *P. gingivalis*. Out of 1232 genes, 765 genes were upregulated, while 467 genes were downregulated in *P. gingivalis*-treated cells (Fig. 3a). Then, the top twenty most enriched Gene ontology (GO) pathway enrichment analysis for differential genes in *P. gingivalis*-infected HSC-4 cells were performed and the regulation of lipid biosynthetic process attracted our attention (Fig. 3b). In order to confirme whether *P. gingivalis* regulates lipid synthesis of OSCC cells, we carried out Nile red staining to evaluate the lipid levels. As shown in Fig. 3c, the levels of cellular lipid droplets were significantly higher in *P. gingivalis*-infected cells than in control cells. To explore whether lipid abundance regulates CSCs properties of OSCC cells, we further treated HSC-4 and SCC-9 cells with Triacsin C, a pan-acyl-CoA synthetase ligase inhibitor, to inhibit neutral lipid synthesis and lipid droplet abundance (Fig. 3c). We found that reduced lipid abundance by Triacsin C significantly suppressed *P. gingivalis*-increased tumorsphere formation capacity (Fig. 3d and Supplementary Fig. 5a) and the expression of CSCs markers (Fig. 3e and Supplementary Fig. 5b). These results confirmed our suspicion that *P. gingivalis* induced the stemness of OSCC cells through regulating lipid synthesis.

### *P. gingivalis* augmented OSCC cells stemness via SCD1-dependent lipid synthesis in vitro

**Fig. 4:**
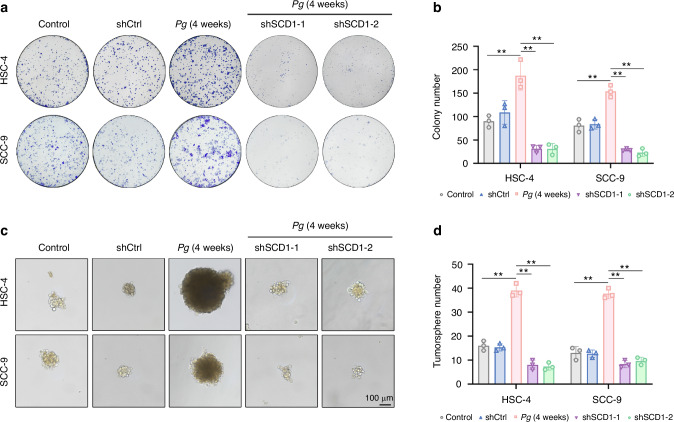
*P. gingivalis* induced OSCC cells to acquire stem-like features by modulating SCD1-mediated lipid synthesis. **a**, **b** Colony formation and quantification showed knockdown of SCD1 decreased *P. gingivalis*-induced the number of cell clones. **c**, **d** Sphere formation and quantification showed knockdown of SCD1 decreased *P. gingivalis*-induced the number and diameter of tumorspheres. Scale bar: 100 μm. *n* = 3. Data are presented as mean ± SD. ** *P* < 0.01

**Fig. 5:**
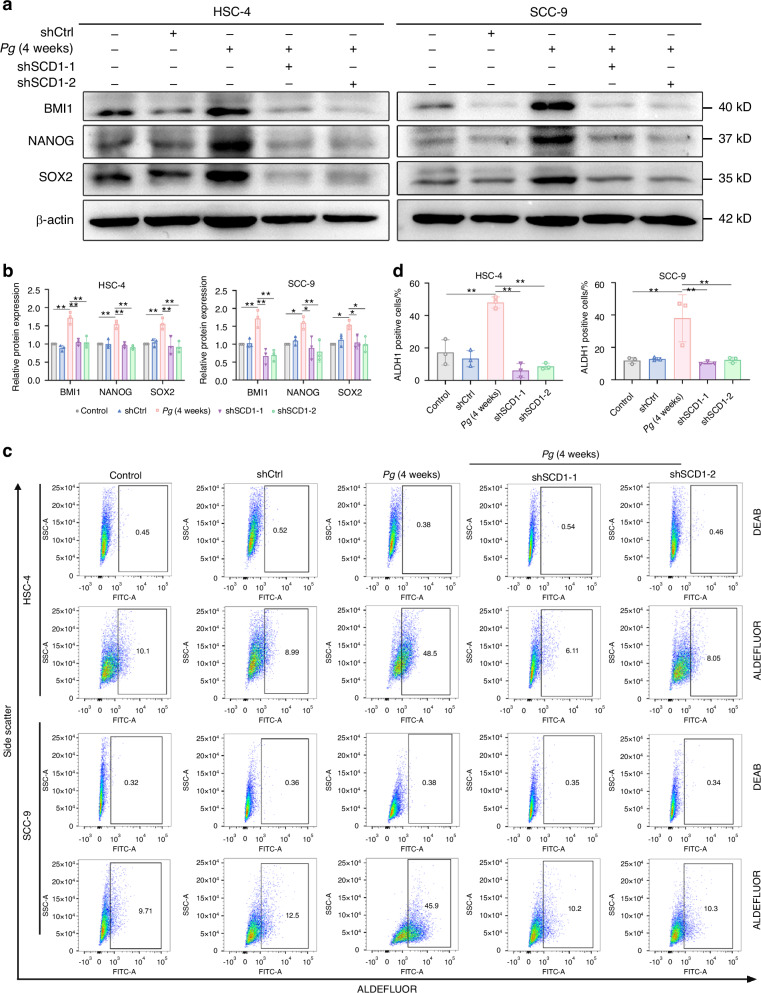
*P. gingivalis* induced OSCC cells to express stemness markers by modulating SCD1-mediated lipid synthesis. **a**, **b** Western blot and quantification showed knockdown of SCD1 suppressed *P. gingivalis*-induced upregulation of BMI1, NANOG, and SOX2. β-actin was used as a housekeeping gene. **c**, **d** Flow cytometry and quantification showed knockdown of SCD1 decreased *P. gingivalis-*increased the proportion of ALDH1^+^ subpopulation of OSCC cells. *n* = 3. Data are presented as mean ± SD. *P < 0.05, ** *P* < 0.01

In cancer cells, FASN, ATP-citrate lyase (ACLY), and SCD1 are considered the key lipogenic enzymes.^33^ Based on the analysis above, we detected the expression of these key enzymes to identify the molecular mechanism by which *P. gingivalis* regulates aberrant lipid metabolism in OSCC cells. Among these enzymes, SCD1 was significantly increased in *P. gingivalis*-infected cells (Supplementary Fig. 5c–e), while other lipogenic enzymes did not change significantly. Hence, we speculated that SCD1 might play a crucial role in *P. gingivalis*-enhanced gain of stem-like features in OSCC cells. To verify this, we efficiently knocked down the expression of SCD1 by shRNA lentivirus (shSCD1-1 and/or shSCD1-2) in OSCC cells and found that SCD1 knockdown decreased the lipid droplets accumulation (Supplementary Fig. 6a), colony formation capacity (Fig. 4a, b), the numbers and size of tumorspheres in both HSC-4 and SCC-9 cells (Fig. 4c, d). Meanwhile, SCD1 depletion significantly abolished *P. gingivalis*-induced upregulation of BMI1, NANOG, and SOX2 at the protein level (Fig. 5a, b). In line with these results, the percentage of the ALDH1^+^ subpopulation was also remarkably suppressed after SCD1 silencing (Fig. 5c, d)). Subsequently, CCK8 assay revealed that *P. gingivalis* enhanced the chemoresistance to cisplatin in OSCC cells, whereas SCD1 suppression impaired cisplatin resistance (Supplementary Fig. 6b). Thus, we proposed that *P. gingivalis* induced OSCC cells to gain stemness via the selective overexpression of SCD1 in vitro. Given the core roles of mitochondria and endoplasmic reticulum in CSCs metabolism, we next performed transmission electron microscopy (TEM) to observe the morphological changes in HSC-4 cells. As shown in Supplementary Fig. 6c, *P. gingivalis*-infected HSC-4 cells appeared morphological features of mitochondria involved a smaller volume and a circular appearance, while mitochondrial swelling and mitochondrial focal cavitation was observed in SCD1-knockdown groups. There was no significant change in endoplasmic reticulum morphological.

Wnt/β-catenin, sonic hedgehog, and notch signaling pathways are known as classical stem cell-related signaling pathways and play a decisive role in tumor stemness.^34^ Subsequently, we detected the expression of critical effectors individually of these signaling pathways and found that *P. gingivalis* infection specially increased the expression of nuclear β-catenin and the phosphorylation status of β-catenin did not change significantly (Supplementary Fig. 7a, b). Similar results were also obtained by immunofluorescence staining (Supplementary Fig. 7c, d). Of note, *P. gingivalis* not only enhanced Shh and Gli1 expression (Supplementary Fig. 7a, b) but also promoted Gli1 nuclear translocation (Supplementary Fig. 7d), while knockdown of SCD1 suppressed this effect. Collectively, these data suggest that *P. gingivalis* promoted lipid synthesis by upregulating SCD1 expression, thereby activating stemness driving factors β-catenin, Shh, and Gli1 to promote OSCC cells to gain stem-like features.

### Silencing of SCD1 reversed *P. gingivalis*-induced stemness and tumorigenesis in vivo

**Fig. 6:**
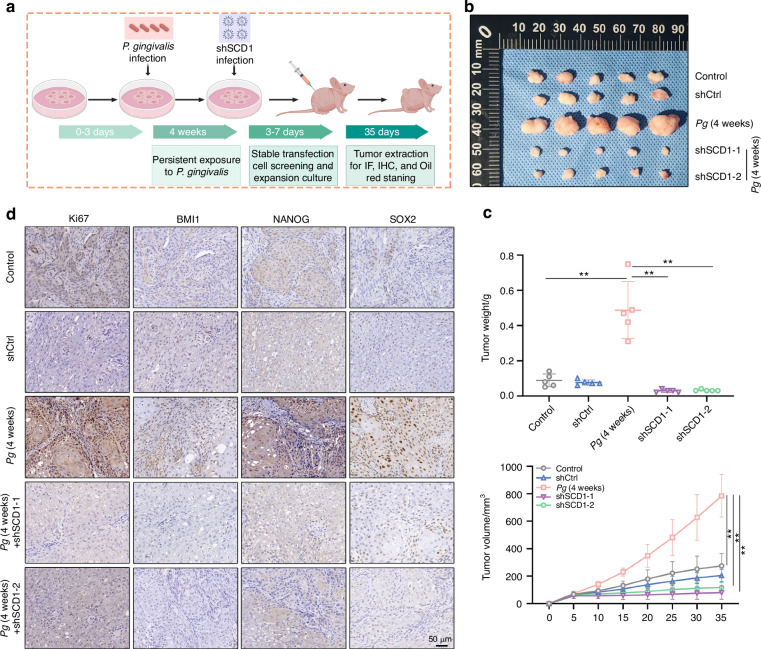
Silencing of SCD1 reversed *P. gingivalis*-induced stemness and tumorigenesis in vivo. **a**, **b** Representative data of the tumors in nude mice under different conditions. **c** Statistical analysis of tumor volumes and weights in the different groups. **d** Representative IHC images of Ki67, BMI1, NANOG, and SOX2 in tumor tissues. Scale bar: 50 μm. *n* = 5. Data are presented as the mean ± SD. ** *P* < 0.01

To further validate its function of SCD1 on OSCC stemness, a nude mouse xenograft model was established (Fig. 6a). As expected, *P. gingivalis* significantly enhanced the tumor-forming ability of HSC-4 cells compared with control group, whereas SCD1 depletion reversed this effect (Fig. 6b). The quantitative results showed *P. gingivalis* group displayed a notably increased tumor volumes and weight, while SCD1 knockdown groups showed smaller volumes and lower weight (Fig. 6c). Correspondingly, immunofluorescence staining results showed the decreased expression of ALDH1 protein level in the SCD1-silenced tumors and the existence of *P. gingivalis* in tumors, which was mostly present around ALDH1 positive area (Supplementary Fig. 8a). The expression level of Ki67, a cell proliferation marker, in the *P. gingivalis* group was significantly higher than that of the control group, indicating that the proliferation of tumor cells in the *P. gingivalis* group was more active and the degree of tumor malignancy was relatively high, which could be reversed by the decrease of SCD1 expression (Fig. 6d and Supplementary Fig. 8b). Moreover, *P. gingivalis*-enhanced expression of SOX2, BMI1, and NANOG in tumor tissues were remarkably reduced by SCD1 knockdown (Fig. 6d and Supplementary Fig. 8b). Additionally, Oil red O staining showed SCD1 knockdown impaired *P. gingivalis*-induced lipid synthesis in tumor tissues (Supplementary Fig. 8c, d). These results demonstrated that *P. gingivalis* promotes OSCC cells stemness and tumorigenesis by SCD1-mediated lipid biosynthesis in vivo.

### SCD1 upregulation in OSCC cells was transcriptionally activated by KLF5

**Fig. 7:**
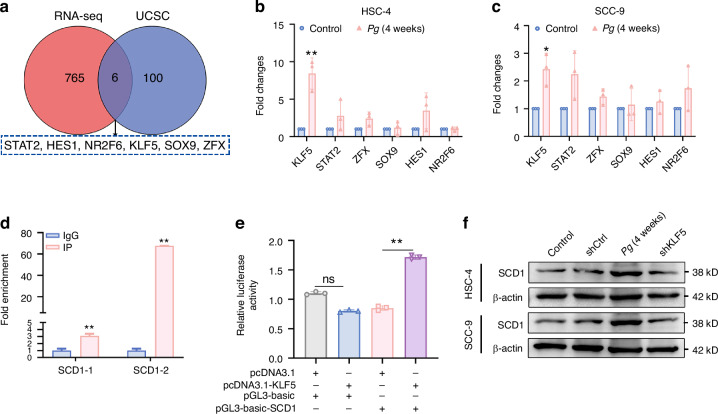
SCD1 upregulation in OSCC cells was transcriptionally activated by KLF5. **a** Venn diagram showing the candidate transcription factor screening process. **b**, **c** qRT-PCR showed *P. gingivalis* specifically promoted the expression of KLF5. **d** ChIP-qPCR showed the binding of KLF5 to the SCD1 promoter region in HSC-4 cells. **e** Dual-luciferase reporter assay showed the binding of KLF5 to the SCD1 promoter region in 293 T cells. **f** Western blot showed knockdown of KLF5 suppressed the protein level of SCD1. β-actin was used as a housekeeping gene. *n* = 3. Data are presented as the mean ± SD. ns: *P* > 0.05, *P < 0.05, ** *P* < 0.01

Given our finding that *P. gingivalis* induced the upregulation of SCD1, which was necessary for OSCC cells stemness acquisition, we further sought to investigate how *P. gingivalis* induced the expression of SCD1. We used the UCSC Genome Browser database (https://genome.ucsc.edu) to identify potential upstream transcription factors that might bind to SCD1, and one hundred potential SCD1-associated transcription factors were found. After overlapping these potential transcription factors (blue circle) with the 765 *P. gingivalis*-upregulated genes with a fold change >1.3 (red circle), we identified six transcription factors (STAT2, NR2F6, HES1, KLF5, SOX9, and ZFX) that might regulate SCD1 (Fig. 7a). Then, we performed the qRT-PCR analysis and found the mRNA level of KLF5 was significantly increased (Fig. 7b, c). Consistently, *P. gingivalis* significantly upregulated the protein level of KLF5 (Supplementary Fig. 9a, b). By performing TCGA OSCC dataset, we showed SCD1 expression positively correlated with KLF5 expression (Supplementary Fig. 9c–h). Predictions by the JASPAR database also identified the presence of possible binding sites of KLF5 in the promoter regions of SCD1 (Supplementary Fig. 9i). To verify this prediction, 3 pairs of primers were designed and synthesized near the binding site with the highest score 5′-GCCCCGCCCA-3′ for ChIP-qPCR verification. The results showed that anti-KLF5 collected more DNA fragments containing the SCD1 promoter region at primers SCD1-1and SCD1-2 (*P* < 0.01), but did not amplification at primer SCD1-3, suggesting that KLF5 was bound to the SCD1 promoter region (Fig. 7d). We further verified the presence of binding between the promoter region of KLF5 and SCD1 in vitro by dual-luciferase reporter assay. The results showed that KLF5 transfected group increased the luciferase activity of pGL3-basic-SCD by almost twofold compared with the pcDNA3.1 (Fig. 7e). These findings indicated that KLF5 was the specific transcription factor of SCD1. Next, we went on evaluating the contribution of KLF5 to the induction of SCD1 of OSCC cells and found that knockdown of KLF5 significantly decreased the protein level of SCD1 (Fig. 7f and Supplementary Fig. 9j). Based on these results, we concluded that KLF5 acted as a transcription activator of SCD1 and was involved in *P. gingivalis-*induced upregulation of SCD1.

### The NOD-like receptor signaling pathway was involved in *P. gingivalis* induced expression of KLF5

**Fig. 8:**
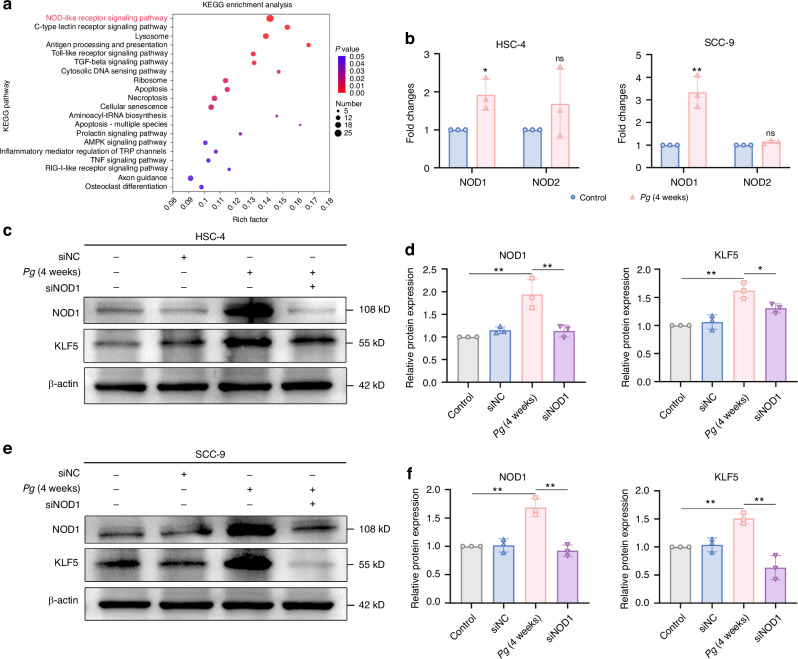
*P. gingivalis* stabilized KLF5 expression via the activation of NOD1. **a** Top twenty most enriched KEGG pathways among significantly upregulated DEGs in HSC-4 cells co-cultured with *P. gingivalis*. **b** qRT-PCR showed *P. gingivalis* promoted the *NOD1* gene expression of OSCC cells. **c**–**f** Western blot and quantification showed silencing of NOD1 decreased the expression of KLF5 in OSCC cells. β-actin was used as a housekeeping gene. *n* = 3. Data are presented as the mean ± SD. ns: *P* > 0.05, *P < 0.05, ** *P* < 0.01

Finally, we assessed how *P. gingivalis* regulated the expression of KLF5. In KEGG pathway analyses of *P. gingivalis*-infected HSC-4 cells, the most enriched pathway was the ‘NOD-like receptor signaling pathway’ (Fig. 8a). Apart from Toll-like receptors (TLRs), nucleotide-binding oligomerization domain (NOD)-containing protein-like receptors (NLRs), a group of receptors that is located in the cytoplasm, has been discovered to recognize invading pathogens and mediate the inflammatory response.^35^ We hypothesized that *P. gingivalis* elevated KLF5 expression, likely through activation of NOD-like receptor signaling pathway. NOD1 and NOD2 are representative members of the NLR family, which functions to stimulate host responses to resist bacterial infection.^36^ Then, we detected the *NOD1* and *NOD2* mRNA levels and found *P. gingivalis* significantly promoted NOD1 expression (Fig. 8b). We subsequently observed repressed KLF5 expression caused by siNOD1 (Fig. 8c–f). Collectively, we propose that *P. gingivalis* promoted SCD1 expression via the NOD1/KLF5 axis.

## Discussion

**Fig. 9:**
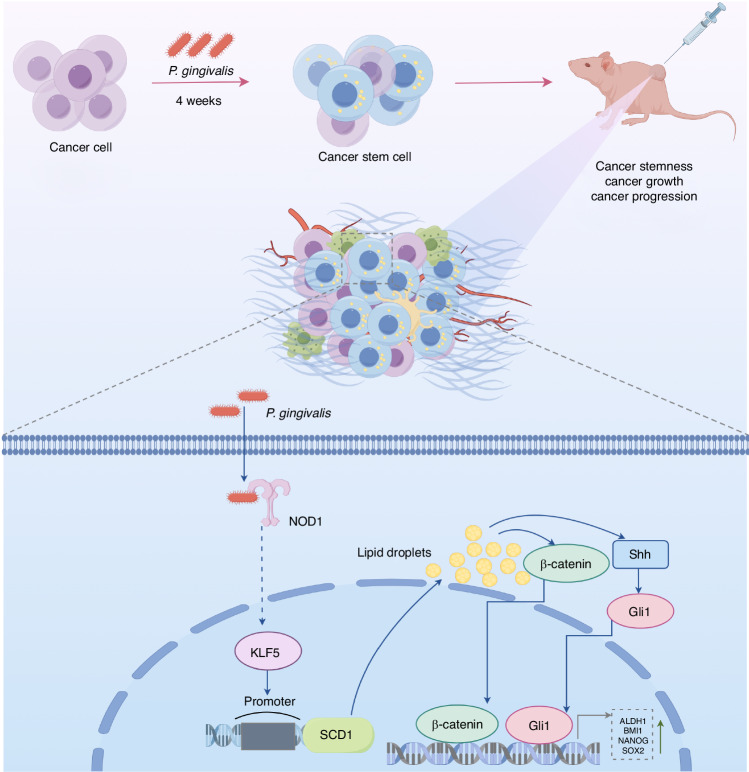
Schematic drawing of the role of *P. gingivalis* in regulating OSCC stemness. The specific molecular mechanism is that *P. gingivalis* promotes OSCC stemness by modulating SCD1-dependent lipid synthesis via NOD1/KLF5 axis. The figure was drawn using Figdraw

Currently, increasing attention is being given to the relationship between microorganisms and cancer cell stemness. In this study, we systematically confirmed a strong association between *P. gingivalis* infection and OSCC cell stemness through the use of clinical tissue samples, a cellular model of persistent infection with *P. gingivalis*, and an in vivo xenotransplantation model. According to high-throughput sequencing and bioinformatics analysis, SCD1-driven lipid synthesis was a pivotal bridge between *P. gingivalis* and OSCC cell stemness. Further studies illustrated that *P. gingivalis* promoted SCD1 expression via the NOD1/KLF5 axis and subsequently activated the stemness driving factors β-catenin, Shh, and Gli1 by regulating the synthesis of intracellular lipid droplets. Thus, for the first time, we proposed a mechanism for the enhanced acquisition of stem-like properties in OSCC cells resulting from *P. gingivalis* infection (Fig. 9).

Through in vitro and in vivo model systems, studies have revealed a role for CSCs in the tumorigenesis of oral cancer. At present, it is widely believed that CSCs originate from mature tumor cells and dedifferentiate into stem cells through modifications in regulatory mechanisms via interactions with the surrounding tumor microenvironment.^37^ In our previous study, we reported that *P. gingivalis* was enriched in OSCC tissues and associated with OSCC progression.^10,11^ However, evidence regarding the association between *P. gingivalis* and OSCC cell stemness has not yet been addressed. In this study, we originally observed that *P. gingivalis* was positively correlated with the expression levels of ALDH1, SOX2, NANOG, and BMI1 in OSCC clinical tissues, whereas there was no correlation with OCT4. This finding was consistent with a previous study in which high OCT4 expression was associated with early-stage OSCC.^38^ For the first time, we suggest that *P. gingivalis* infection promotes OSCC cell stemness acquisition by regulating the expression of the aforementioned CSC markers, distinguishing *P. gingivalis* from other bacteria with regard to stemness induction.^16^ *P. gingivalis* has been shown to involve in tissue colonization by expressing a variety of virulence factors.^39^ To further explore the bacterial components involved in the regulation of stemness markers, heat-inactivated *P. gingivalis*, gingipains, and LPS were applied and the results showed that they could not induce the expression of stemness markers, which identify that live whole *P. gingivalis* was required for stemness induction. A previous study has indentified the differences in gene expression when *P. gingivalis* grew either in planktonic or in biofilms states.^40^ Therefore, it is needed to explore the effect of *P. gingivalis* within biofilm growth on the stemness of OSCC cells, which will be an important part of our future work.

Emerging evidence suggests that microorganisms promote malignant tumor progression by manipulating metabolic reprogramming.^41^ Herein, through high-throughput sequencing and bioinformatics analysis, we found significant enrichment of multiple lipid metabolism pathways, which supports our hypothesis. Previous studies have demonstrated that alterations in lipid metabolism are required for the maintenance of CSC properties in different tumor types.^42^ Importantly, the specific process of lipid biosynthesis has been shown to be an effective target for impairing CSCs.^43^ The aberrant expression of key lipogenesis factors contributes to metabolic alterations.^44^ In colorectal cancer cell lines, the abnormal lipid accumulation induced by *F. nucleatum* was shown to be associated with increased FASN protein expression.^16^ Intriguingly, our results revealed that aberrant lipid droplet accumulation in OSCC cells caused by persistent exposure to *P. gingivalis* was dependent on the expression of SCD1, indicating that SCD1 plays a major role in *P. gingivalis*-induced lipid metabolism alterations. SCD1 is considered a biochemical hallmark of cancer cells and modulates the growth, stemness, and metastasis of multiple tumors.^45,46^ Nevertheless, the extent to which SCD1 mediates *P. gingivalis*-induced OSCC cell stemness has not been explored. Establishment of a cellular model of persistent infection with *P. gingivalis*, along with corroboration by an in vivo xenotransplantation model, demonstrated SCD1 acting as a key regulator in oral CSCs maintenance and that targeting SCD1 may be a prospective therapeutic option for OSCC. Notably, multiple studies have highlighted the central role of the ER and mitochondria in CSCs metabolism and stemness maintenance.^47,48^ In the present study, we observed a distinct mitochondrial morphology in HSC-4 cells exposed to different treatments. The smaller volume and perinuclear localization pattern of the mitochondria in *P. gingivalis*-infected cells were consistent with the mitochondria in leukemia stem cells.^49^ Interestingly, we also observed mitochondrial swelling and focal cavitation in SCD1-depleted cells, suggesting that SCD1 may also play a role in maintaining the unique mitochondrial morphology of *P. gingivalis*-infected cells.

Notch, Shh, and Wnt signals are evolutionarily conserved pathways, and the persistent activation of those pathways serves a crucial role in maintaining the stemness and tumorigenicity of CSCs.^34^ Our study revealed that *P. gingivalis* could activate β-catenin, resulting in the translocation of β-catenin from the cytoplasm to the nucleus, where it regulates the expression of specific target genes. A previous study indicated that the inhibition of SCD1 significantly reduced the gene expression of the Wnt signaling pathway in colon cancer cells.^50^ Consistent with that work, we found that SCD1 silencing significantly restrained the activation of β-catenin in OSCC cells infected with *P. gingivalis*. In addition, for the first time, our study revealed that the overexpression of Shh and Gli1 induced by *P. gingivalis* is regulated by SCD1 in OSCC cells and that SCD1 inhibition led to the suppression of Shh and Gli1. Of note, *P. gingivalis* induced Gli1 transferred to nucleus, suggesting the involvement of SCD1 in activating Shh signaling. Therefore, we concluded that SCD1 promoted OSCC cell stemness through activating the stemness driving factors β-catenin, Shh, and Gli1.

In further investigations of the regulatory mechanisms of SCD1 during *P. gingivalis* infection, using the UCSC Genome Browser database and validation assays, we determined that KLF5 might be an upstream factor of SCD1. KLF5 is an important transcription factor that regulates various cancerous processes including proliferation, migration, stemness, and metastasis, and is highly expressed in various cancer tissues.^51,52^ Specifically, KLF5 has been shown to modulate fatty acid synthesis by activating the nuclear receptor PPARG in esophageal adenocarcinoma.^53^ Although some studies have revealed the important role of KLF5 in the regulation of lipid metabolism, whether KLF5 can regulate lipid metabolism by affecting SCD1 expression has not been reported. Through TCGA database analysis, we found that there was a positive correlation between KLF5 and SCD1 expression in OSCC tissues. In addition, our findings indicate for the first time that KLF5 acts as a transcription activator, directly binding to the SCD1 promoter region and subsequently activating its expression. Taken together, these findings support our hypothesis that the expression of SCD1 is regulated by KLF5 under *P. gingivalis* infection. Nevertheless, the binding sites of KLF5 and SCD1 remain unclear, which will be the subject of future work.

Previous studies have suggested that the cell type-specific host immune response to *P. gingivalis* infection is closely linked to the activation of pattern recognition receptors (PRRs).^54^ NODs are a group of intracellular pattern-recognition proteins that recognize invading pathogens and induce host responses to limit bacterial infection.^55^ In the present study, *P. gingivalis* upregulated the expression of NOD1 but not NOD2. NOD1 mainly recognizes γ-D-glu-meso-diaminopimelic acid-containning peptidoglycans (PGNs) in the cell wall of gram-negative bacteria and is highly expressed in several malignancies such as cervical cancer, colon cancer, and head and neck squamous cell carcinoma.^56–58^ However, a specific role for NOD1 in *P. gingivalis*-infection induced stemness acquisition of OSCC cells has not been identified. Here, we identified that NOD1 silencing reversed the upregulation of KLF5 expression caused by *P. gingivalis*, which indicated that NOD1 played a non-negligible role in response to *P. gingivalis*-infection. KLF5 is considered as an unstable protein that can be ubiquitinated and degraded. Several deubiquitinases, such as ubiquitin-specific peptidase 38 (USP38), deubiquitinase (DUB) BAP1, affect the protein stability of KLF5 through deubiquitination.^59,60^ A previous study reported that NOD1 controlled the expression of deubiquitinase CYLD in response to Klebsiella infection and that NOD1 knockdown abolished the upregulation of CYLD expression.^61^ Therefore, we supposed that the *P. gingivalis*-mediated stabilization of KLF5 expression might be dependent on NOD1 activation, which will be explored in our follow-up study.

In summary, our results uncovered that *P. gingivalis* promoted OSCC cells to gain stem-like properties by modulating SCD1-dependent lipid biosynthesis via the NOD1/KLF5 axis, which ultimately promoted OSCC growth and progression. Our findings provide new insights into the potential mechanisms of OSCC progression under *P. gingivalis* infection and suggest that SCD1 is a molecular target that can be exploited for OSCC treatment in the future.

## Materials and methods

### Patients and specimen collection

A total of 30 OSCC specimens used for immunohistochemical (IHC) and quantitative real-time PCR analysis were collected from the Hospital of Stomatology of China Medical University (Shenyang, China) between 2021 and 2022. The inclusion criteria were patients who had confirmed primary OSCC without other malignancies and were undergoing tumorectomy. The pateints’ clinical data is shown in Supplementary Table 1. The tumor grading and TNM classification were completed by two independent pathologists. The use of OSCC samples for this study was approved by the Affiliated Stomatological Hospital of China Medical University Institutional Review Board (K2021010) and all patients gave informed consent.

### Immunohistochemical staining

Formalin-fixed paraffin-embedded (FFPE) tumor tissues were serially sectioned into 4 μm sections. Tissue paraffin sections were de-waxed, rehydrated, blocked, and incubated with the primary antibodies, listed in Supplementary Table 2, overnight at 4 °C. Subsequently, each section was incubated with polyperoxidase-anti-rabbit/mouse IgG (ZSGB-BIO, China) at room temperature for 30 min, stained with diaminobenzidine, and counterstained with haematoxylin.

### Cell and bacteria culture

The OSCC cell lines HSC-4 and SCC-9 were originally derived from a patient with tongue cancer and respectively obtained from the Japanese Collection of Research Bioresources Cell Bank (JCRB; Shinjuku, Japan) and American Type Culture Collection (ATCC, Manassas, USA). HSC-4 cells were cultured in DMEM/high glucose (Gibco, USA) supplemented with 10% fetal bovine serum (FBS). SCC-9 cells were cultured and passaged in DMEM/Ham’s F-12 medium with 10% FBS and 400 ng/mL hydrocortisone (Sigma-Aldrich, USA). *P. gingivalis* ATCC 33277 (American Type Culture Collection) were cultured on brain heart infusion (Difco Laboratories, MI, USA) agar plates at 37 °C in an anaerobic chamber (80% N_2_, 10% H_2_, and 10% CO_2_). For all the experiments in our study, cells were challenged with *P. gingivalis* at a multiplicity of infection (MOI) of 10 for 4 weeks. Subsequently, long-term infection cells lysed with sterile distilled water for 40 min were collected, inoculated on agar plates with appropriate dilution and cultured anaerobically for 7 days.

### Cell viability assay

HSC-4 and SCC-9 cells were seeded into 96-well plates at a density of 3 000 cells per well overnight. Subsequently, cells were treated with *P. gingivalis* for 24, 48, and 72 h. 10 μL CCK-8 reagent (SparkJade, China) was added to each well. After 2 h of incubation at 37 °C in the dark, the plates were determined at 450 nm by a microplate reader (Tecan, Mechelen, Belgium).

As for drug resistance assay, cells were exposed to different concentrations of cisplatin (0, 2.5, 5, 10, 20, and 40 μmol/L) for 48 h.

### kFluor488 Click-iT EdU assay

Cell proliferation was measured using EdU detection kit (5-Ethynyl-2’-deoxyuridine) (KeyGENBioTECH, China) according to the producer’s instructions. OSCC cells were seeded at 5 × 10^4^ per well on 24-well plates. Subsequently, 10 μmol/L EdU labeling media was added to culture cells for another 2 h and then the cells were stained with 1× Click-iT EdU staining mix for 30 min in the dark. These cells were subsequently counterstained with Hoechst33342 for 20 min and imaged by a fluorescence microscope (200×) (Nikon DS-Ri2).

### Cell migration and invasion assay

For wound-healing assay, a wound was created and incubated in serum-free medium. Cell motility was determined by measuring the average scratch width change. For transwell invasion assay, Matrigel (BD Biosciences, USA) was used to precoated the upper chamber of transwell (pore size, 8 μm; Corning, NY, USA) for 1 h at 37 °C and the cells were seeded into the upper chambers (1 × 10^5^ cells per well) in 200 μL serum-free medium. After incubation for 48 h, cells were fixed in 4% paraformaldehyde and stained with 0.1% crystal violet dye. Then, the chambers were washed and photographed under the microscope (Nikon DS-Ri2).

### Colony formation assay

1 000 cells were seeded in the six-well plate and incubated for 12 days. The cells were fixed with 4% paraformaldehyde for 20 min and stained with 0.1% crystal violet for 15 min. Then, excess stain was washed with water and number of colonies more than 50 cells was counted.

### Sphere formation assay

2 000 cells were plated in the ultra-low attachment 6-well plate (Corning, USA). Cells were grown in a serum-free DMEM or DMEM/F12 medium supplemented with 2% B27 supplement (ThermoFisher Scientific, USA), 20 ng/mL human EGF (Novoprotein, China), and 20 ng/mL human bFGF (Novoprotein, China). The medium was changed every 2–3 days. After incubation for 12 days, spheres larger than 100 μm in diameter were counted under Nikon DS-Ri2 microscope.

### Quantitative real-time PCR

Total RNA was extracted from OSCC tissues or OSCC cells using TRIzol reagent (ThermoFisher Scientific, Waltham, MA, USA) and synthesized cDNA was prepared with a PrimeScriptTM RT Reagent Kit (Takara Bio Inc., Dalian, China). Quantitative real-time PCR (qRT-PCR) analysis were performed with a 7500 Real-time PCR system using SYBR™ Green Master Mix (Takara Bio Inc., Dalian, China). The primers used in this study are listed in Supplementary Table 3. Fold changes were analyzed using the comparative 2^−ΔΔCt^ determination method.

### Western blot analysis

The protein concentrations were quantified by BCA protein assay kit (Beyotime Biotechnology, China). Nuclear and Cytoplasmic Protein Extraction Kit (Beyotime Biotechnology, China) was used to separate nuclear and cytoplasmic proteinaccording to the manufacturer’s instructions. The primary antibodies used in this study were listed in Supplementary Table 2. Secondary antibodies include HRP-linked anti-mouse IgG or anti-rabbit IgG (Absin, China). Protein bands were visualized using Tanon 5200 (Tanon, China) and signals were quantified using ImageJ software.

### ALDH1 assay

The ALDEFLUOR^TM^ kit (STEMCELL, Canada) was applied to determine the ratio of ALDH enzymatic activity. 5 × 10^5^ cells were re-suspended in the ALDEFLUOR assay buffer containing 1 μmol/L ALDH1 substrate BAAA, and then 500 µL of the cell suspension was immediately transferred into the control tube and incubated with 5 µL of ALDH inhibitor diethylaminobenzaldehyde (DEAB) for calculating background. After incubated at 37 °C for 45 min in the dark, cells were washed twice and the samples were detected using a flow cytometry (FACS, BD, USA).

### Nile red staining

The cells were washed twice with PBS and fixed in 4% paraformaldehyde for 20 min. Then the cells were incubated in Nile red staining solution (1 mg/L, Solarbio, China) in the dark for 20 min at 37 °C. Finally, the immunostained samples were counterstained with DAPI (Beyotime Biotech. Co.). Images were captured an Olympus FV-1000 confocal microscope (Tokyo, Japan).

### Transcriptome sequencing (RNA-Seq) analysis

Total RNA from HSC-4 cells and long-term *P. gingivalis*-infected HSC-4 cells was isolated using TRIzol (Takara, Japan). After detected by NanoDrop, the samples were applied by ILLumina Hiseqxten/NovaSeq6000 for transcriptomic high-throughput sequencing (Magorbio Ltd, Shanghai, China). The RNA-seq analysis was performed on the Majorbio Cloud Platform (www.majorbio.com). Differential expression analysis was performed using DESeq2 packages. The criteria including |Log_2_FC| ≥1.3, *P* < 0.05, and FDR < 0.05 were applied to select the significantly differential mRNAs. Gene Ontology (GO) enrichment and KEGG analysis were completed to identify the biological functions of the differential mRNAs and the pathways they participate in.

### Lentivirus and RNA interference

Lentivirus containing SCD1 short hairpin (sh) RNA, lentivirus containing KLF5 short hairpin (sh) RNA, and empty vector lentiviral particles were synthesised by Genechem (Shanghai, China). The shRNA sequences are listed in Supplementary Table 4. Puromycin (2 μg/mL, MCE, Shanghai, China) was used to selecte the stably transfected cells. Specific siRNA against NOD1 and siNC were designed and synthesised by KELBiotech (Shanghai, China) and listed in Supplementary Table 5. Lipofectamin®3000 reagent (L3000008, Invitrogen) was used to transfect cells with siRNA according to the manufacturer’s instructions.

### Transmission electron microscopy

After different treatment, HSC-4 cells were collected and fixed in 2.5% glutaraldehyde for 2 h at 4 °C. Then, the samples were cut into ultra-thin slices and the ultrastructure was acquired using a transmission electron microscope (H7650, Hitachi, Tokyo, Japan).

### Immunofluorescent staining

Tissue paraffin sections were de-waxed, rehydrated, and blocked. Then, the sections were incubated with hybridization buffer containing *P. gingivalis* oligonucleotide probe (5′-GGTTTTCACCATCAGTCATCAACA-3′) in a dark humid chamber at 48 °C for 2 h and then incubated with the ALDH1 overnight at 4 °C. For OSCC cells, the samples were fixed with 4% PFA and treated with 0.2% TritonX-100. Subsequently, primary antibodies were incubated overnight at 4 °C. The samples were immunostained with Alexa Fluor 594-conjugated secondary antibodies (Proteintech, China) for 2 h and counterstained with DAPI (Beyotime Biotech. Co.). Images were captured with a laser-scanning confocal fluorescence microscope (OLYMPUS FV3000, Japan).

### Mouse xenografts

The experiment was approved by the Ethics Committee of the School and Hospital of Stomatology at China Medical University. Six-week-old male BALB/c nude mice were purchased from Si Pei Fu (Beijing, China). The mice were maintained in a specific-pathogen free facility with a 12 h:12 h light:dark cycle at 21–23 °C. Two hundred microliters of diluted Martrigel containing 3 × 10^6^ tumor cells were injected subcutaneously into the mice. The size of the tumor was measured every 5 days and tumor volumes were calculated using the digital caliper (width^2^ × length)/2. After 35 days, mice were euthanized and the tumor tissues were removed and analyzed.

### ChIP-qPCR assay

The BeyoChIP™Assay Kit (Beyotime Biotechnology) was performed according to manufacturer’s instructions. In brief, *P. gingivalis*-infected HSC-4 cells were cross-linked with 1% formaldehyde for 10 min. Glycine was used to terminate the cross-linking reaction. Then, the cells were collected, lysed and sheared into 200–1 000 base pair fragments on ice, which were immunoprecipitated with anti-KLF5 antibody (Proteintech) or rabbit IgG (Proteintech) overnight at 4 °C. The fold-enrichment of KLF5 binding on specific region of the SCD1 promoter was detected by qRT-PCR. The primer sequences were listed in Supplementary Table  6.

### Dual luciferase reporter assay

293 T cells, obtained from ATCCC, were seeded in 24-well plate and transfected with corresponding plasmids (pcDNA3.1, pGL3-basic, KLF5-pcDNA3.1, pGL3-basic-SCD, PRL-TK) with Lipofectamin® 3000. After culture for 48 h, the relative luciferase activity was determined with a Dual-Luciferase Reporter Assay System (11402ES60, Yeasen Biotechnology, China) according to the manufacturer’s manuals.

### Statistical analysis

Statistical analyses were performed by GraphPad Prism 9.0. One-way ANOVA analysis was used for comparison among multiple groups (Tukey’s multiple comparisons test was used for multiple comparison afterwards), and independent sample *t* test was used for comparison between two groups. Pearson correlation analysis was applied for correlation analysis. All experiments were repeated three times. *P*-value < 0.05 was considered statistically significant.

## Supplementary information

Supplementary Files
