# Supplementary material for: Porphyromonas gingivalis potentiates stem-like properties of oral squamous cell carcinoma by modulating SCD1-dependent lipid synthesis via NOD1/KLF5 axis
Source: Int J Oral Sci. 2025 Feb 28;17:15. doi: 10.1038/s41368-024-00342-8 (PMC11868650; doi:10.1038/s41368-024-00342-8)
Supplement: Supplementary file 1 — Supplementary Files [file 41368_2024_342_MOESM1_ESM.docx]

Supplementary information for

***Porphyromonas gingivalis* potentiates stem-like properties of oral squamous cell carcinoma by modulating SCD1-dependent lipid synthesis via NOD1/KLF5 axis**

Wenli Zang^1^, Fengxue Geng^1^, Junchao Liu^1^, Zengxu Wang^2^, Shuwei Zhang^1^, Yuchao Li^1^, Ze Lu^1^, Yaping Pan^3*^

1. Department of Periodontics, School and Hospital of Stomatology, China Medical University, Liaoning Provincial Key Laboratory of Oral Disease, Shenyang 110002, Liaoning, China
2. Department of Oral Maxillofacial-Head and Neck Surgery, School and Hospital of Stomatology, China Medical University, Liaoning Provincial Key Laboratory of Oral Disease, Shenyang 110002, Liaoning, China

* Corresponding author:

Yaping Pan

1. Department of Periodontics and Oral Biology, School and Hospital of Stomatology, China Medical University, Liaoning Provincial Key Laboratory of Oral Disease

Nanjing North St. 117, Heping District, Shenyang 110002, Liaoning, China

Tel: 86-24-31927706, Fax: 86-24-31927188

E-mail: yppan@cmu.edu.cn

**This file includes:**

Supplementary Figures and Tables

**Supplementary Figures**

**
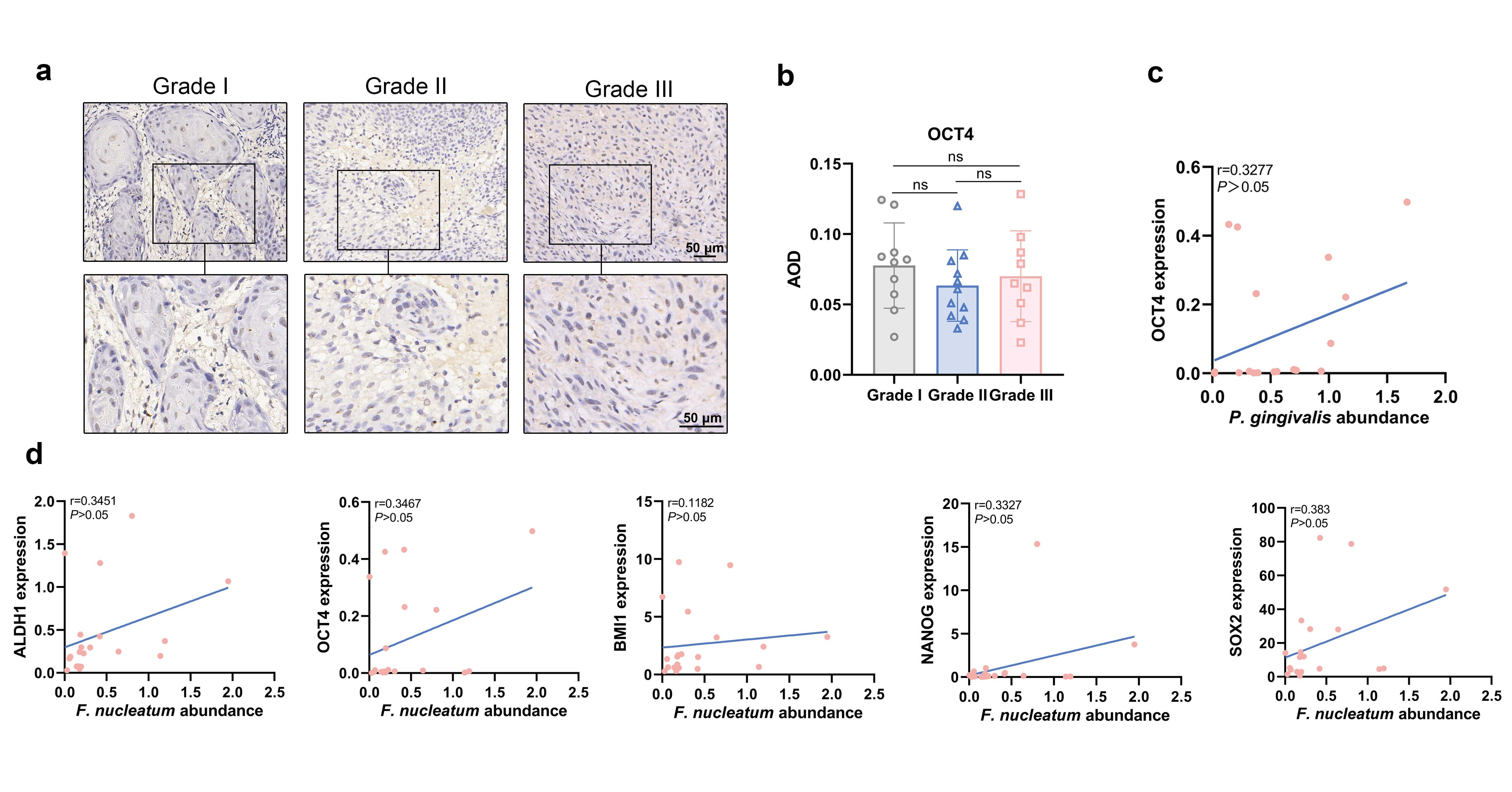
**

Fig. S1 **a** Representative IHC images of OCT4 in OSCC samples. Scale bar: 50 μm. **b** Quantitative analysis of OCT4 in OSCC samples. *n* = 30. Data are presented as mean ± SD. ns: *P >* 0.05. **c** The correlation between *P. gingivalis* abundance and the expression of OCT4 was analyzed with Pearson correlation analysis. *n* = 20. **d** The correlations between *F. nucleatum* abundance and the expression of ALDH1, OCT4, BMI1, NANOG, and SOX2 were analyzed with Pearson correlation analysis. *n*=20.

**
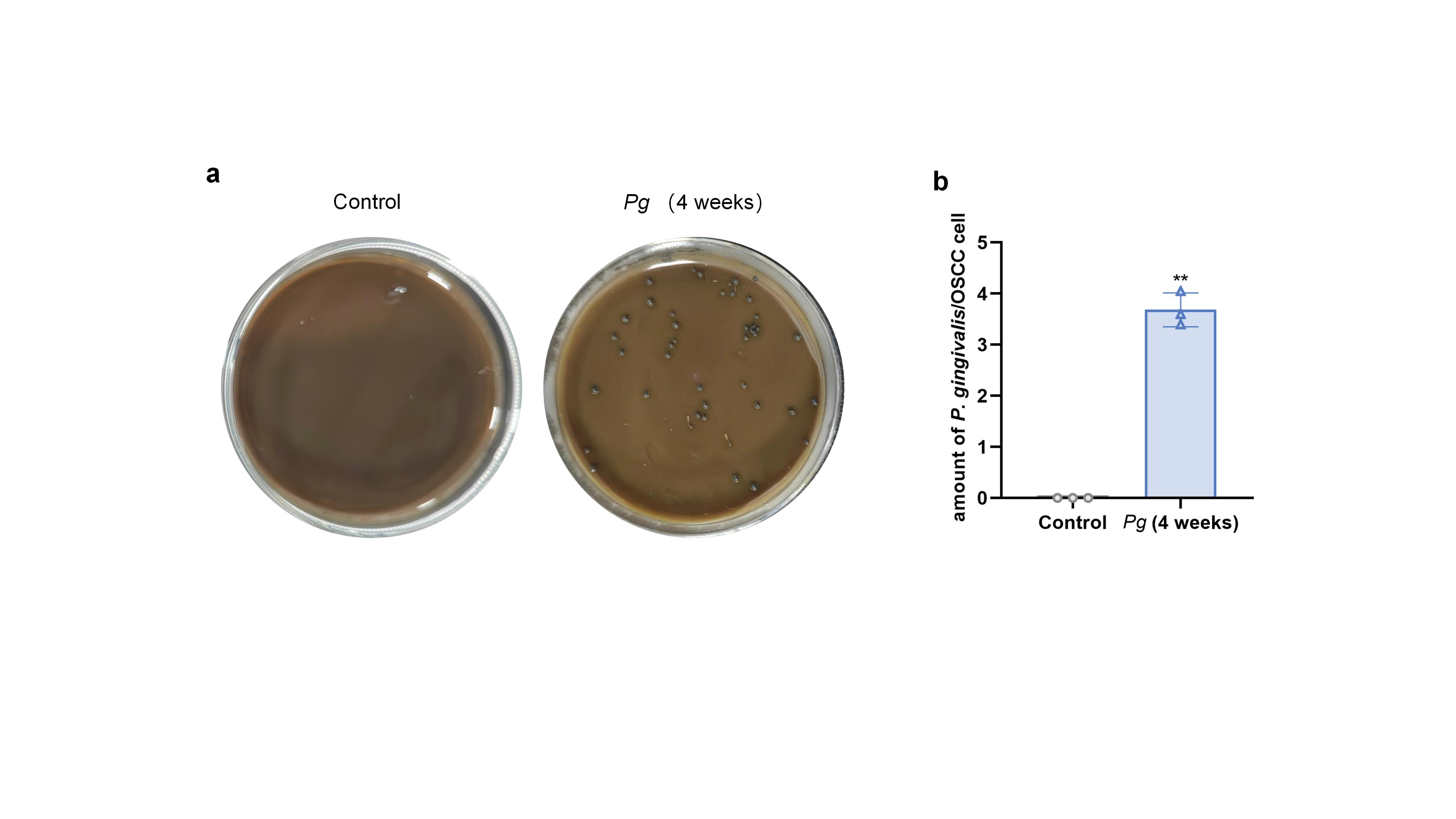
**

Fig. S2. *P. gingivalis* could remain viable after 4 weeks incubation with the OSCC cells in culture. **a, b** Representative images of colonies and quantification indicated that *P. gingivalis* could remain viable during long-term infection. *n*=3. Data are presented as mean ± SD. ** *P* < 0.01.


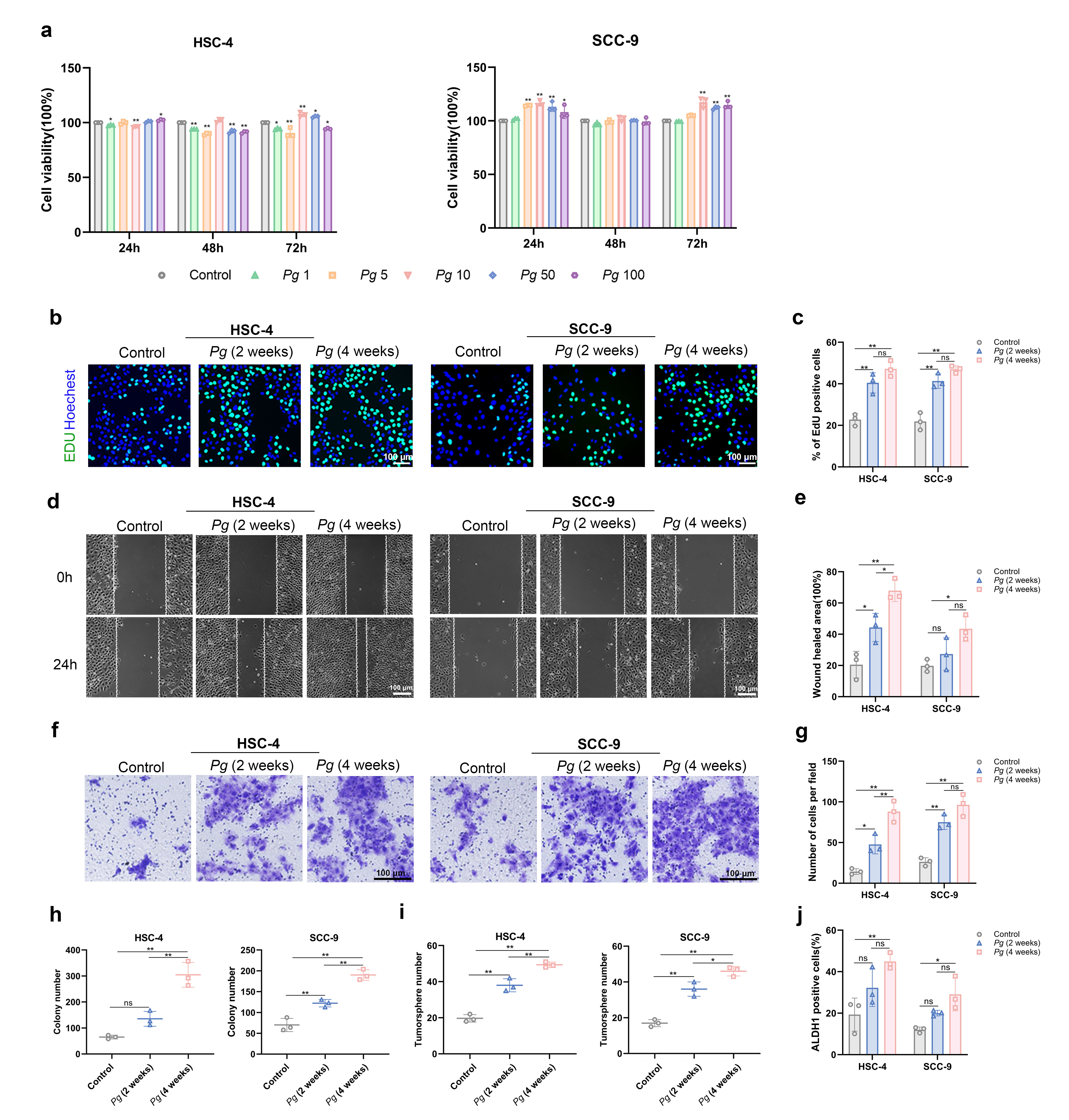


Fig. S3. Effects of the *P. gingivalis* on the malignant biological behaviors of OSCC cells. **a** CCK8 showed *P. gingivalis* at MOI 10 specifically promoted the viability of OSCC cells in a time-dependent manner. **b, c** EdU incorporation assay showed persistent infection with *P. gingivalis* at MOI 10 promoted the proliferation of HSC-4 and SCC-9 cells. **d, e** Wound-healing assay and quantification showed the ability of cell migration was accelerated after *P. gingivalis* infection. **f, g** Transwell assay and quantification showed *P. gingivalis* promoted the invasion of OSCC cells. **h** Colony formation quantification showed *P. gingivalis* increased the number of cell clones. **i** Sphere formation quantification showed *P. gingivalis* increased the number of tumorspheres. **j** Flow cytometry quantification showed *P. gingivalis* increased the proportion of ALDH1^+^ subpopulation of HSC-4 and SCC-9 cells. Scale bar: 100 μm. *n*=3. Data are presented as mean ± SD. ns *P* > 0.05, **P* < 0.05, ** *P* < 0.01.


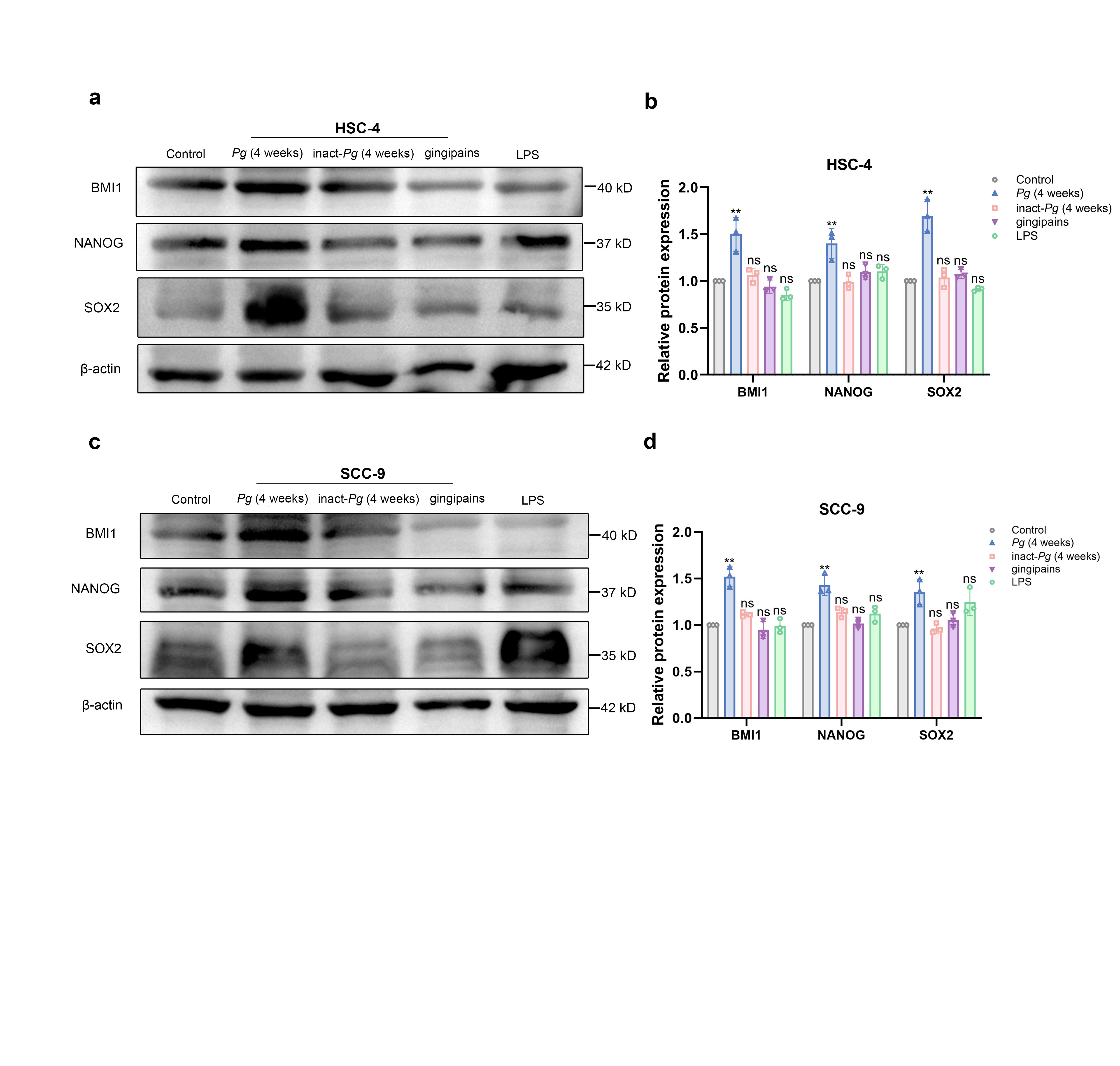


Fig. S4. *P. gingivalis* induced the expression of stemness markers of OSCC cells may depend on bacterial activity. **a-d** Western blot and quantification showed the expression of BMI1, NANOG, and SOX2 in OSCC cells under different treatment. Data are presented as the mean ± SD. *n*=3. ns *P* > 0.05, ** *P* < 0.01.


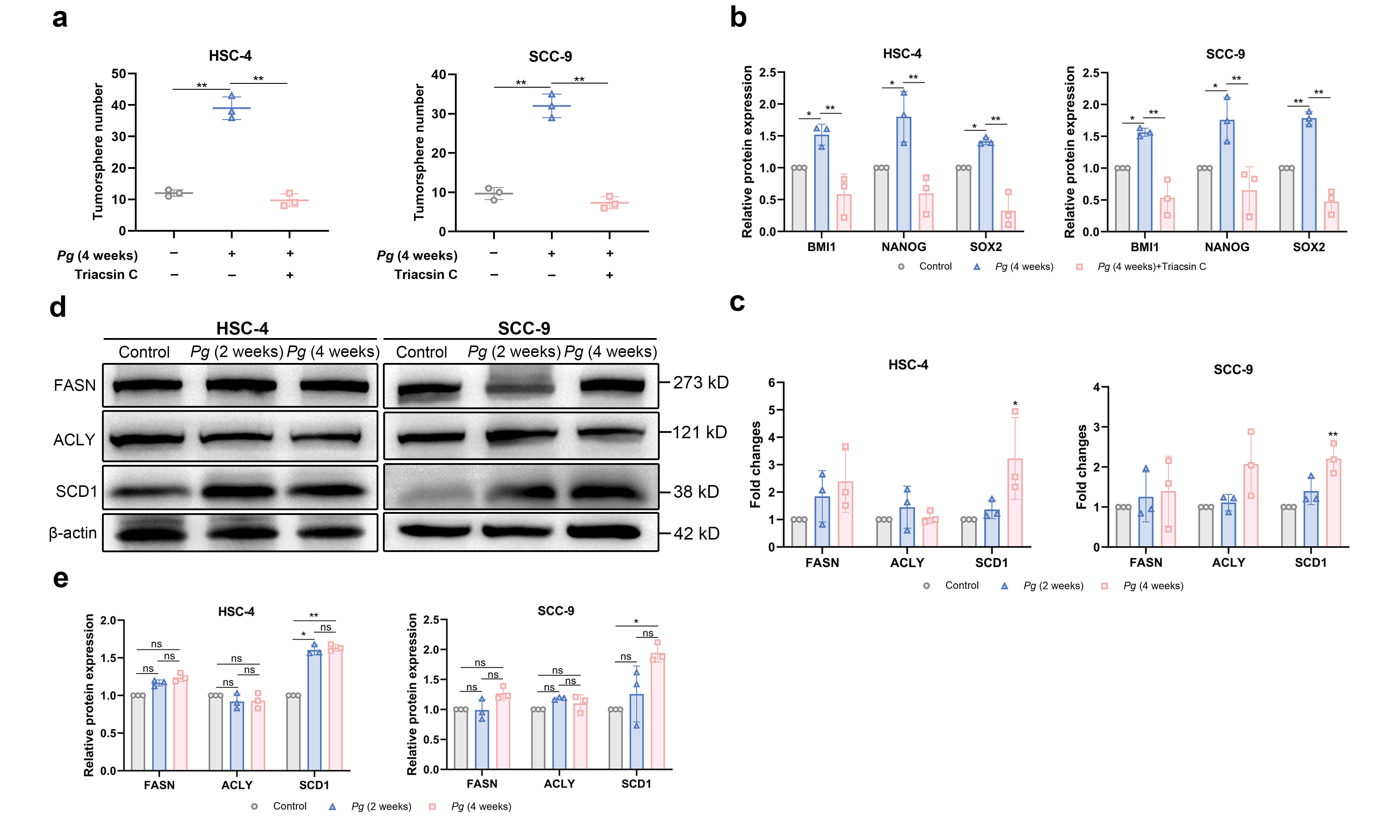


Fig. S5. *P. gingivalis* induced OSCC cells to acquire stem-like features by regulating lipid synthesis and promoted SCD1 expression. **a** Sphere formation quantification showed Triacsin C decreased *P. gingivalis*-induced the number of tumorspheres. **b** Western blot quantification showed Triacsin C significantly suppressed *P. gingivalis*-induced upregulation of BMI1, NANOG, and SOX2. **c** qRT-PCR showed the gene expression of FASN, ACLY, and SCD1 after *P. gingivalis* infection. **d, e** Western blot and quantification showed the protein level of FASN, ACLY, and SCD1 after *P. gingivalis* infection. β-actin was used as a housekeeping gene. *n*=3. Data are presented as the mean ± SD. ns *P* > 0.05, **P* < 0.05, ** *P* < 0.01.


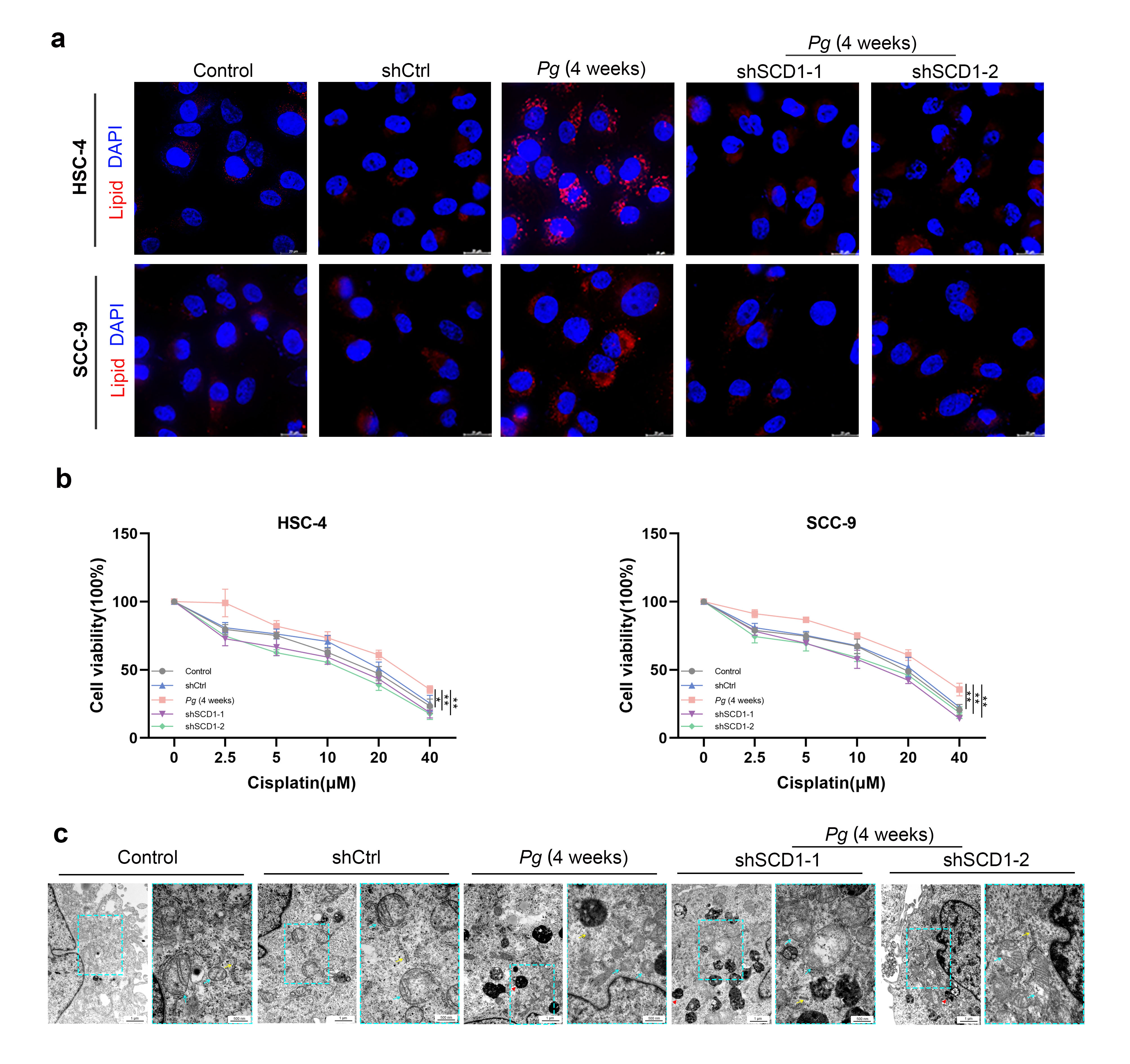


Fig. S6. *P. gingivalis* induced OSCC cells chemoresistance by modulating SCD1-mediated lipid synthesis. **a** Representative images of Nile red staining showed intracellular lipid droplets content in each group. Scale bar: 20 μm. **b** CCK8 showed SCD1 suppressed *P. gingivalis*-induced chemoresistance in OSCC cells. **c** Representative images of HSC-4 cells ultrastructure after different treatment. (Yellow arrows indicated endoplasmic reticulum; blue arrows indicated mitochondria; red triangle indicated *P. gingivalis*). *n*=3. Data are presented as the mean ± SD. **P* < 0.05, ** *P* < 0.01.


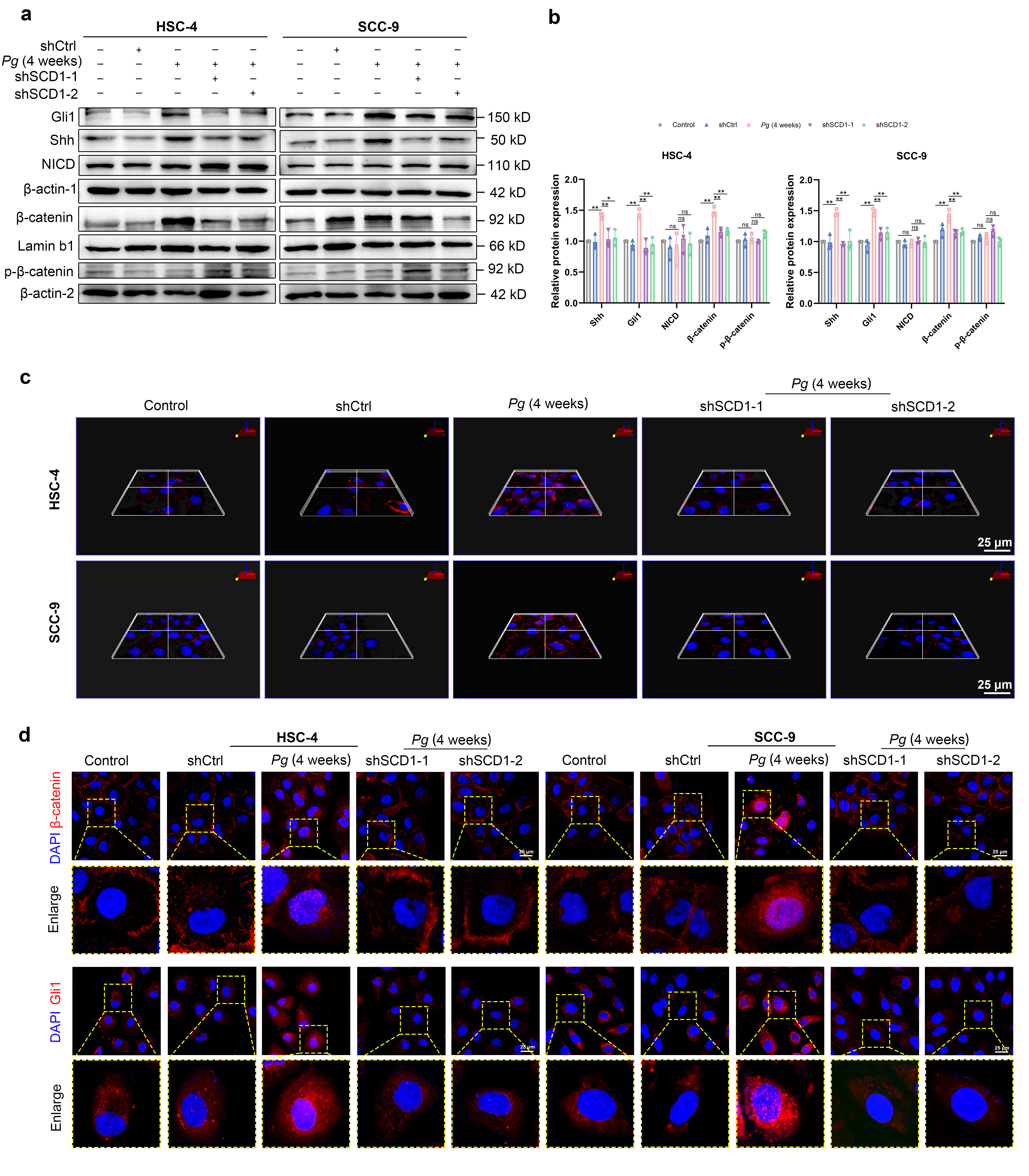


Fig. S7. SCD1 promoted OSCC cells stemness by activating β-catenin, Shh, and Gli1 expression. **a,** **b** Western blot and quantification showed knockdown of SCD abolished *P. gingivalis*-induced upregulation of β-catenin, Shh, and Gli1. β-actin and lamin b1 were used as housekeeping genes. **c** Immunofluorescent staining showed the representative 3D images of β-catenin subcellular localization in OSCC cells. **d** Immunofluorescent staining showed the representative images of β-catenin and Gli1 subcellular localization in OSCC cells. Scale bar: 25 μm. *n*=3. Data are presented as the mean ± SD. ns *P* > 0.05, **P* < 0.05, ** *P* < 0.01.


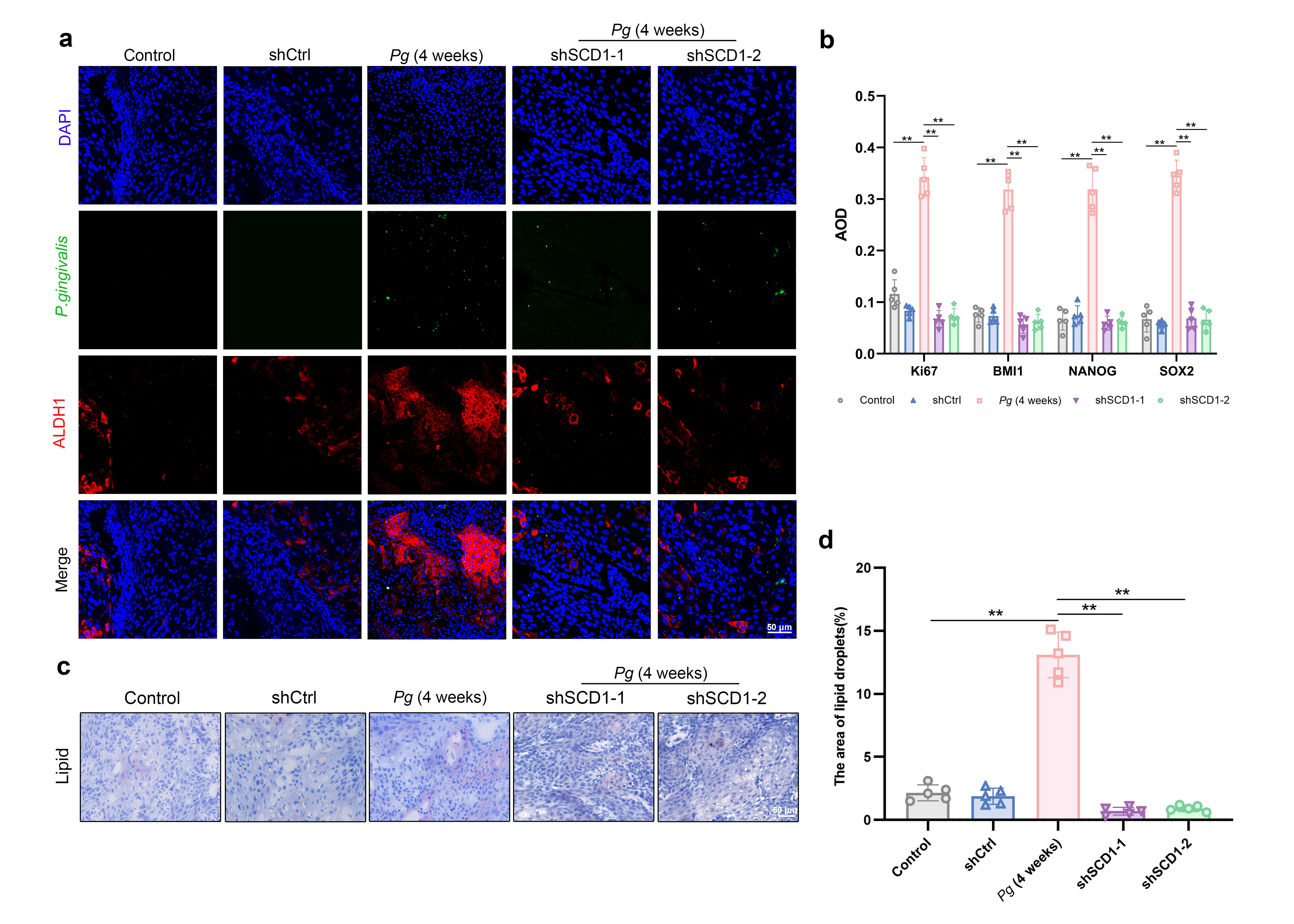


Fig. S8. Silencing of SCD1 reversed *P. gingivalis*-induced stemness in vivo. **a** Immunofluorescent staining showed the representative images of ALDH1 and *P. gingivalis* expression in nude mice tumor tissues. **b** Quantitative analysis of Ki67, BMI1, NANOG, and SOX2 expression in nude mice tumor tissues. **c** Representative Oil Red O staining images of lipid in tumor tissues. **d** Quantitative analysis of lipid droplets in nude mice tumor tissues. Scale bar: 50 μm. *n*=5. Data are presented as the mean ± SD. ** *P* < 0.01.


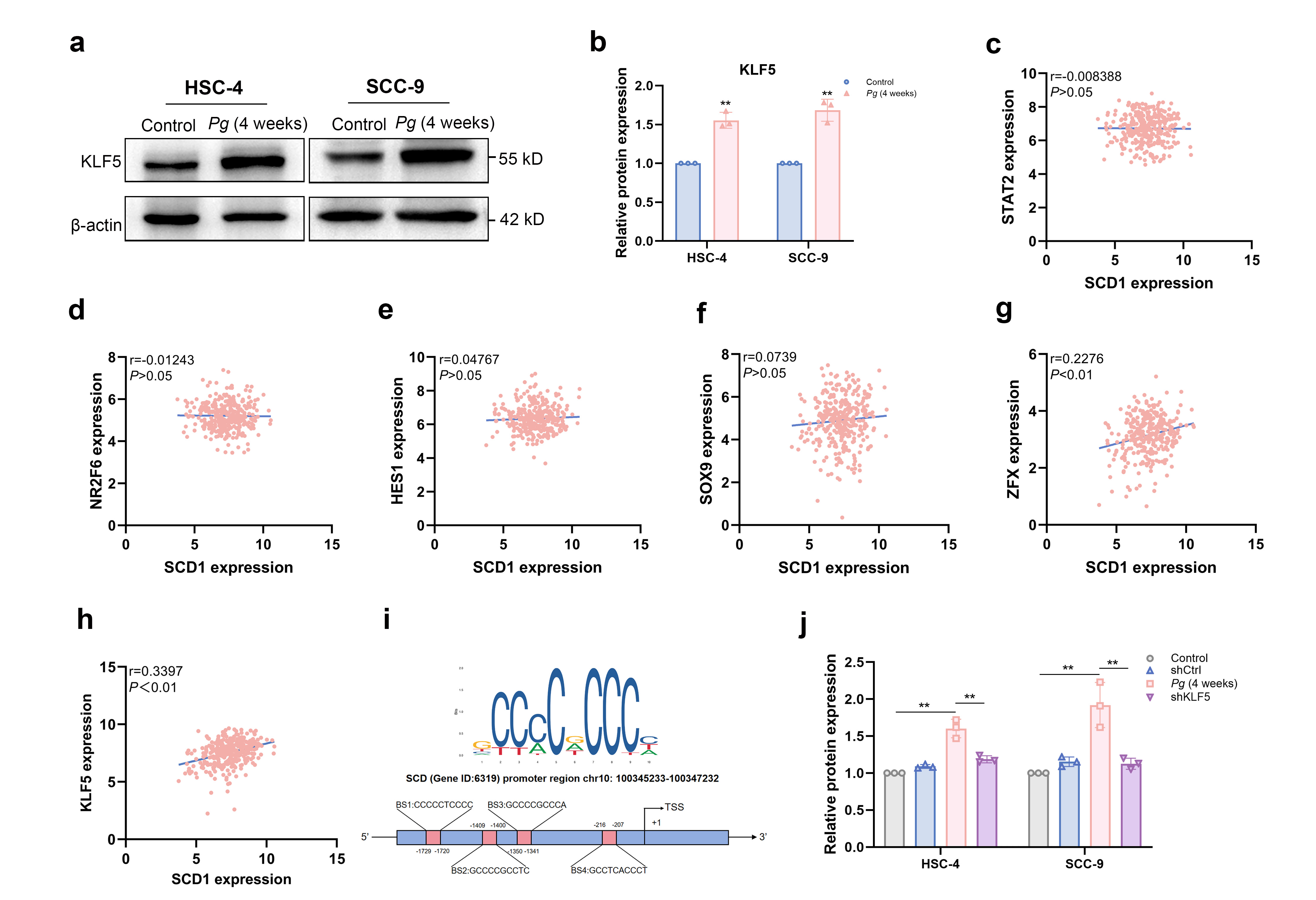


Fig. S9. SCD1 upregulation in OSCC cells was transcriptionally activated by KLF5. **a, b** Western blot and quantification showed *P. gingivalis* upregulated the protein level of KLF5. **c-h** TCGA database showed the correlation between SCD1 expression and STAT2, NR2F6, HES1, SOX9, ZFX, KLF5 expression in OSCC samples. *n*=327. **i** The presence of possible binding sites of KLF5 in the promoter regions of SCD1. **j** Western blot quantification showed knockdown of KLF5 suppressed the protein level of SCD1. *n*=3. Data are presented as the mean ± SD. ** *P* < 0.01.

Supplementary Table 1

**Table 1 Clinical characteristics of 30 OSCC patients**

| Characteristics | Case | % |
| --- | --- | --- |
| Age (years) |  |  |
| ≤60 | 17 | 56.7% |
| >60 | 13 | 43.3% |
| Sex |  |  |
| Male | 18 | 60.0% |
| Female | 12 | 40.0% |
| TNM stage |  |  |
| Ⅰ-Ⅱ | 22 | 73.3% |
| Ⅲ-Ⅳ | 8 | 26.7% |
| Hitological grade |  |  |
| Grade I | 10 | 33.3% |
| Grade II | 11 | 36.7% |
| Grade III | 9 | 30.0% |

Supplementary Table 2

**Table 2 The antibodies used in our study**

| Antibody | Item No | Company | Concentration |
| --- | --- | --- | --- |
| BMI1 | 66161-1-Ig | Proteintech | 1:100(IHC)/1:1000(WB) |
| SOX2 | 3579 | Cell Signaling Technology | 1:200(IHC)/1:1000(WB) |
| NANOG | 14295-1-AP | Proteintech | 1:200(IHC)/1:1000(WB) |
| SCD1 | 28678-1-AP | Proteintech | 1:1000(WB) |
| ACLY | 15421-1-AP | Proteintech | 1:1000(WB) |
| FASN | D162701 | Sangon Biotech | 1:1000(WB) |
| Shh | 20697-1-AP | Proteintech | 1:1000(WB) |
| β-catenin | 8480 | Cell Signaling Technology | 1:200(IF)/1:1000(WB) |
| NICD | 4147 | Cell Signaling Technology | 1:1000(WB) |
| Gli1 | 66905-1-Ig | Proteintech | 1:200(IF)/1:1000(WB) |
| ALDH1 | 54135 | Cell Signaling Technology | 1:200(IHC)/1:1000(WB) |
| OCT4 | ab200834 | Abcam | 1:150(IHC) |
| Antibody | Item No | Company | Concentration |
| β-actin | 66009-1-Ig | Proteintech | 1:1000(WB) |
| Lamin b1 | ab133741 | Abcam | 1:1000(WB) |
| NOD1 | A1246 | Abclonal | 1:1000(WB) |

Supplementary Table 3

**Table 3 Primer sequences used in this study**

| Gene | Sense |
| --- | --- |
| *P. gingivalis* Forward | 5'-AGGCAGCTTGCCATACTGCG-3' |
| *P. gingivalis* Reverse | 5'-ACTGTTAGCAACTACCGATG-3' |
| *F. nucleatum* Forward | 5'-GTGGTAAGCTGCGATAAGCCTAG-3' |
| *F. nucleatum* Reverse | 5'-CGTGTTCCATGTTACTTGGGAGCA-3' |
| *Universal bacteria* Forward | 5'-CCATGAAGTCGGAATCGCTGA-3' |
| *Universal bacteria* Reverse | 5'-GCTTGACGGGCG GTGT-3' |
| *ALDH1* Forward | 5'-CCGTGGCGTACTATGGATGC-3' |
| *ALDH1* Reverse | 5'-GCAGCAGACGATCTCTTTCGAT-3' |
| *SOX2* Forward | 5'-CGAACCATCTCTGTGGTCT-3' |
| *SOX2* Reverse | 5'-GTGTCAACCTGCATGGC-3' |
| *NANOG* Forward | 5'-TTTGTGGGCCTGAAGAAAACT-3' |
| *NANOG* Reverse | 5'-AGGGCTGTCCTGAATAAGCAG-3' |
| *BMI1* Forward | 5'-CGTGTATTGTTCGTTACCTGGA-3' |
| *BMI1* Reverse | 5'-TTCAGTAGTGGTCTGGTCTTGT-3' |
| *OCT4* Forward | 5'-GCAAGCCCTCATTTCACC-3' |
| *OCT4* Reverse | 5'-CCATCACCTCCACCACCT-3' |
| *GAPDH* Forward | 5'-GAAGGTGAAGGTCGGAGTC-3' |
| *GAPDH* Reverse | 5'-GAAGATGGTGATGGGATTTC-3' |
| *STAT2* Forward | 5'-CCCTCCCTGTGGTGATTATTT-3' |
| *STAT2* Reverse | 5'-AGAACTGCTGGTTCTGAAGG-3' |
| *KLF5* Forward | 5'-ACACCAGACCGCAGCTCCA-3' |
| *KLF5* Reverse | 5'-TCCATTGCTGCTGTCTGATTTGTAG-3' |
| *SOX9* Forward | 5'- CAAGAAGGACCACCCGGATT-3' |
| *SOX9* Reverse | 5'-AAGATGGCGTTGGGGGAGAT-3' |
| Gene | Sense |
| *NR2F6* Forward | 5'-TCTAGCTCCTATACCACCACCA-3' |
| *NR2F6* Reverse | 5'-TCGTCTCCAACTTATCTCCTCC-3' |
| *HES1* Forward | 5'-TCAACACGACACCGGATAAAC-3' |
| *HES1* Reverse | 5'-GCCGCGAGCTATCTTTCTTCA-3' |
| *ZFX* Forward | 5'-TTGCTGAAATCGCTGACGAAG-3' |
| *ZFX* Reverse | 5'-GCAATCGGCATGAAGGTTTTGAT-3' |
| *NOD1* Forward | 5'-CCAAGTTCGTGCTGTGCTATGC -3' |
| *NOD1* Reverse | 5'-GGCTGCCCAGGCTCTCATTG-3' |
| *NOD2* Forward | 5'-CACGGTGAAAGCGAATGGATTGG-3' |
| *NOD2* Reverse | 5'-GAGGAAGCGAGACTGAGCAGAC-3' |

Supplementary Table 4

**Table 4 The shRNA sequences**

| NO. | Target Seq |
| --- | --- |
| SCD-RNAi1 | CGTCCTTATGACAAGAACATT |
| SCD-RNAi2 | CTACGGCTCTTTCTGATCATT |
| SCD-RNAi3 | CCCACCTACAAGGATAAGGAA |
| KLF5-RNAi | CGGATCTAGATATGCCCAGTT |

Supplementary Table 5

**Table 5 The siRNA sequences**

|  | Sense（5'-3'） | Antisense（5'-3'） |
| --- | --- | --- |
| siRNA-1 | GCGAAGAGCUGACCAAAUATT | UAUUUGGUCAGCUCUUCGCTT |
| siRNA-2 | GUCCAAAGCCAAACAGAAATT | UUUCUGUUUGGCUUUGGACTT |
| siRNA-3 | GCGUUCAGGUCGAAAGCUUTT | AAGCUUUCGACCUGAACGCTT |
| siNC | UUCUCCGAACGUGUCACGUTT | ACGUGACACGUUCGGAGAATT |

Supplementary Table 6

**Table 6 The SCD1 primer sequences**

|  | Sense（5'-3'） | Antisense（5'-3'） |
| --- | --- | --- |
| SCD1-1 | GCAGGCACACACACACCA | CCCACCACTAACATCTCCGT |
| SCD1-2 | ACACACACACCAGCCAGCC | CCACCACTAACATCTCCGTCC |
| SCD1-3 | ACCAGCCAGCCTGTGTGT | ATCTCCGTCCCGTCTTCC |
